# Comparison of normalization methods for Illumina BeadChip HumanHT-12 v3

**DOI:** 10.1186/1471-2164-11-349

**Published:** 2010-06-02

**Authors:** Ramona Schmid, Patrick Baum, Carina Ittrich, Katrin Fundel-Clemens, Wolfgang Huber, Benedikt Brors, Roland Eils, Andreas Weith, Detlev Mennerich, Karsten Quast

**Affiliations:** 1 Boehringer Ingelheim Pharma GmbH & Co. KG, Birkendorfer Str. 65, 88397, Biberach/Riss, Germany; 2 EMBL-EBI, Wellcome Trust Genome Campus, Hinxton, Cambridge CB10 1SD, UK; 3 Theoretical Bioinformatics Department, German Cancer Research Center (DKFZ), Im Neuenheimer Feld 280, 69120 Heidelberg, Germany; 4 Department of Bioinformatics and Functional Genomics, Institute of Pharmacy and Molecular Biotechnology (IPMB) and BioQuant, University of Heidelberg, 69120 Heidelberg, Germany; 5 Boehringer Ingelheim Pharmaceuticals Inc., Ridgefield, CT 06877, USA

## Abstract

**Background:**

Normalization of microarrays is a standard practice to account for and minimize effects which are not due to the controlled factors in an experiment. There is an overwhelming number of different methods that can be applied, none of which is ideally suited for all experimental designs. Thus, it is important to identify a normalization method appropriate for the experimental setup under consideration that is neither too negligent nor too stringent. Major aim is to derive optimal results from the underlying experiment. Comparisons of different normalization methods have already been conducted, none of which, to our knowledge, comparing more than a handful of methods.

**Results:**

In the present study, 25 different ways of pre-processing Illumina Sentrix BeadChip array data are compared. Among others, methods provided by the BeadStudio software are taken into account. Looking at different statistical measures, we point out the ideal versus the actual observations. Additionally, we compare qRT-PCR measurements of transcripts from different ranges of expression intensities to the respective normalized values of the microarray data. Taking together all different kinds of measures, the ideal method for our dataset is identified.

**Conclusions:**

Pre-processing of microarray gene expression experiments has been shown to influence further downstream analysis to a great extent and thus has to be carefully chosen based on the design of the experiment. This study provides a recommendation for deciding which normalization method is best suited for a particular experimental setup.

## Background

Analysing gene expression using microarrays is a well established method [1]. Many different technologies have been developed, of which the most advanced are Affymetrix GeneChip [2] and Illumina Sentrix BeadChip arrays [3]. These high throughput technologies allow the parallel quantification of a large number of transcripts. It is well known in the microarray community that normalization has to be performed to minimize systematic effects that are not constant between different samples of an experiment and that are not due to the factors under investigation (e.g. treatment, time).

Several studies comparing different normalization methods have already been conducted, many of them focusing on Affymetrix chips [4-7], others on Illumina chips [8-12], and only very few have been conducted focusing on both technologies [13,14]. To our knowledge, so far no analysis has been published comparing a large number of different normalization methods for the Illumina BeadChip Technology and only very few studies [8] that took the normalizations offered by BeadStudio into account and compared them to other established normalization methods. Optimal selection of a normalization method depends very heavily on the nature of the experiment. In this regard factors like comparability and quality of single runs play a major role. It has been shown that the normalization method used may influence further downstream analysis to a great extent [6] and thus has to be carefully chosen based on the actual data.

Here we present a strategy for an in depth evaluation of normalization methods aiming at identifying the most appropriate one for a given data set. Our study compares established normalization methods available in the R environment to those offered by BeadStudio software. It focuses on the HumanHT-12 v3 Expression BeadChip, yet the underlying principles are directly transferable to other technologies measuring gene expression. Analyses described here provide the basis for the Phenocopy project (Baum *et al*., Phenocopy - a strategy to qualify chemical compounds during Hit-to-Lead and/or Lead Optimization, submitted 2010). Within this project, the aim is to predict mode of action as well as off-target effects of compounds based on gene expression data. To do so, HaCaT cells were treated with TGF-β as well as seven different compounds in seven dosages and with six siRNAs. Gene expression was measured after 2, 4, and 12 hours using Illumina's Human HT-12 v3 Expression BeadChips [15]. In addition we performed qRT-PCRs using the TaqMan^® ^technology to measure the quantitative abundance of eight genes at three time points that are known to be deregulated [11,14]. In order to identify the normalization method best suited for the experimental design of the Phenocopy project, we, in total, compared 25 different ways of normalization and analysed different statistical aspects of the data.

## Results and Discussion

**Figure 1:**
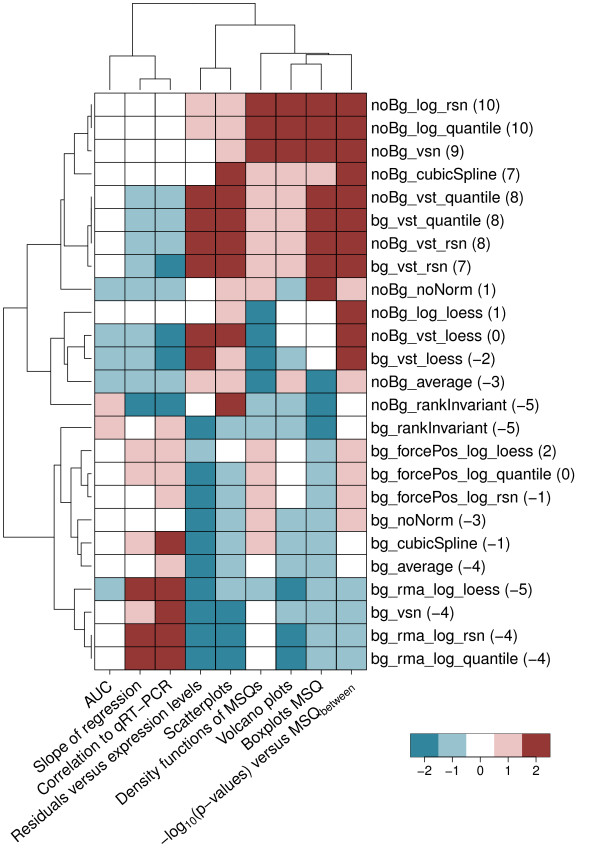
**Heatmap of quality scores assigned for the different pre-processing methods**. Displayed are the quality scores for the different pre-processing methods given for the analyses conducted. Quality scores range from -2 (bad) to 2 (good). The values in parentheses display the sum over the single quality scores for the respective pre-processing procedures. Based on this sum, the pre-processing method finally used to normalize the Phenocopy data has been chosen. Manhattan distance and complete linkage were used for clustering by applying an adjusted heatmap.2() function as implemented in the gplots package [36]. Methods evaluating the bias (slope of regression, correlation to qRT-PCR) are clearly separated from methods evaluating the variance. Pre-processing procedures that perform best based on the sum over quality scores are located at the top of the heatmap.

Expression data was pre-processed in 25 different ways (Figure 1). We focused on analysing the TGF-β stimulated and control samples measured at three time points (2 h, 4 h, 12 h) in four replicates. Generally speaking, first either background normalization from BeadStudio [16] (bg_*) or no background modification (noBg_*) has been applied. In a next step, the data was transformed using either log_2_-transformation (log) or variance-stabilizing transformation (vst) [9]. Since BeadStudio's background normalization can lead to negative values, the data had to be transformed to contain only positive values by using either the background correction of rma [4] or forcePos [17] to be able to apply log_2_-transformation. In a last step, the data was normalized using quantile, loess, or rsn [17] normalization. Alternatively, the transformation steps were skipped and vsn [18] or the normalization methods supplied by BeadStudio (average, rankInvariant, cubicSpline) are used for normalization.

Different pre-processing methods were evaluated by analysing the variance of the resulting gene expression intensities via various statistical measures. Some of these have already been used in other studies [5]. In addition to the investigation of the actual expression intensities, fold changes derived from resulting gene expression intensities were compared to fold changes based on quantitative measurements of RNA abundance as determined by qRT-PCR. Thereby, it is possible to evaluate the pre-processing methods with respect to their bias.

Pre-processing methods were scored from -2 to 2 based on how well they match the required criteria for the different analyses described in this section. As it is difficult to clearly categorize the methods based on the examined measures, the final decision of which score to assign to some extent stays subjective. However, it is unambiguously possible to separate better pre-processing methods from worse. A complete overview of the scores assigned and the final ranking is given in Figure 1.

### Analyses of variance based on expression measurements

One basic assumption of gene expression pre-processing methods is that the majority of genes do not change their expression under different conditions. Additionally, expression intensities of replicates should be very similar compared to the expression of transcripts between differently treated sample groups. Based on these principles, we looked at different statistical measures to identify the method best suited for our dataset with respect to variance.

#### Distribution of F-test statistics

**Figure 2:**
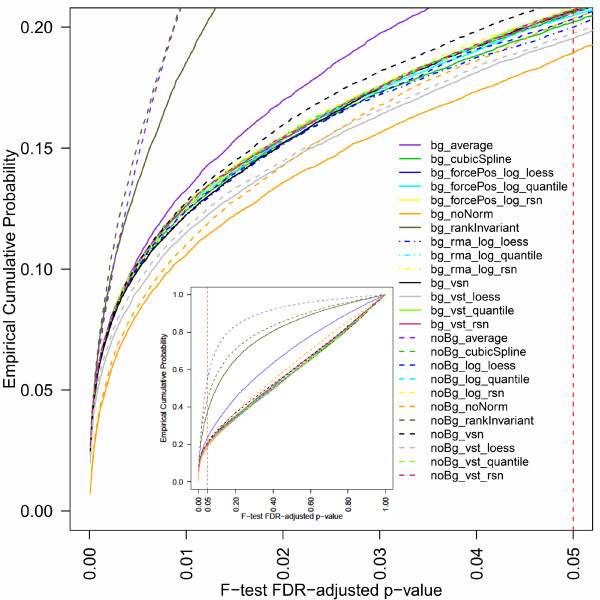
**Cumulative Distribution Functions of F-test p-values**. Cumulative Distribution Functions (CDFs) of FDR-corrected F-test p-values were calculated based on the gene expression measured for untreated HaCaT cells after 2, 4, and 12 hours. Displayed are the results obtained for the different pre-processing methods used. The vertical red dashed line indicates the commonly chosen p-value cut-off of 0.05. The insert displays the obtained results over the whole range of values from 0 to 1 on both axes.

A good normalization method should minimize the variation within a treatment group. Furthermore, the variation within a treatment group should be smaller than the variation between groups. The F-statistic is a typical measurement to compare the variation between replicates to the variation between conditions or treatment groups [19,20]. Results for the F-statistic based on the gene expression measured for the untreated HaCaT cells after 2 h, 4 h, and 12 h are displayed in Figure 2. Four BeadStudio normalization methods (noBg_average, noBg_rankInvariant, bg_rankInvariant, bg_average) show cumulative distribution functions that are clearly above those obtained based on all other pre-processing methods. Applying neither background correction nor any normalization method (noBg_noNorm) results in a data set producing less adjusted p-values < 0.02 than other pre-processing methods. With decreasing significance of the adjusted p-values more pre-processing methods produce fewer p-values of higher significance than noBg_noNorm. Based on the data set used, we expect only a small subset of the transcripts to be significantly deregulated. Since for bg_noNorm compared to other pre-processing methods the fewest genes would be detected as being differentially expressed, i.e. showing a relatively high variation between compared to within group variability, this method seems to provide the best results. The remaining pre-processing methods perform relatively similar and equally well.

#### P-values against variance between groups

**Figure 3:**
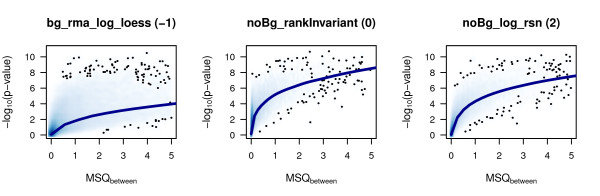
**-log_10_(p-values) against MSQ_between _where MSQ_between _≤ 5**. MSQ_between _was calculated based on the gene expression measured for the three sample groups analyzed, namely untreated HaCaT cells after 2, 4, and 12 hours. Results of three exemplary pre-processing methods of different quality are shown. bg_rma_log_loess exhibits the most unfavourable behaviour of the three. The p-values show a high variability over the whole range of MSQ_between _and even for small MSQ_between _values there are many relatively high -log_10_(p-values). Though for noBg_rankInvariant the p-values show less variability in general, especially for small MSQ_between _values there are even more high -log_10_(p-values). In contrast, noBg_log_rsn exhibits less varying p-values and does not assign as many small p-values to low MSQ_between _regions. The blue line represents a loess-curve fitted to the values. This curve takes uniformly larger values for noBg_rankInvariant and noBg_log_rsn than for bg_rma_log_loess indicating, on average, smaller p-values for the same MSQ_between _value. Thus, in Figure 1, quality values of -1, 0, and 2 are assigned to bg_rma_log_loess, noBg_rankInvariant, and noBg_log_rsn, respectively. For an overview of all different normalization methods and their quality scores, see Additional file 1 and Figure 1.

Assuming a stable variance over the within group measurements, the bigger the variance between the groups, the bigger the respective -log_10_(p-value) should be. When plotting these parameters, an appropriate normalization method should result in smoothly increasing values with not too much scattering around the fitted curve. Figure 3 displays the -log_10_(p-value) against the respective variance between the control groups at time points 2 h, 4 h, and 12 h for three of the pre-processing methods, an overview over all results is given in Additional file 1. Normalizations reflecting the described properties are for example noBg_cubicSpline, noBg_log_rsn, noBg_vst_rsn, and noBg_vsn. All of the normalizations performed on rma background corrected data as well as bg_vsn display a relatively high -log_10_(p-value) for a relatively high proportion of low between group variability values leading to a high scattering of observations in these regions. Using, for example, the rank invariant normalization of BeadStudio (noBg_rankInvariant) the p-values for the low between group variability tend to be relatively small. This could lead to an overestimation of differentially expressed genes when filtering solely based on p-values.

#### Boxplots of MSQ_between _and MSQ_within_

**Figure 4:**
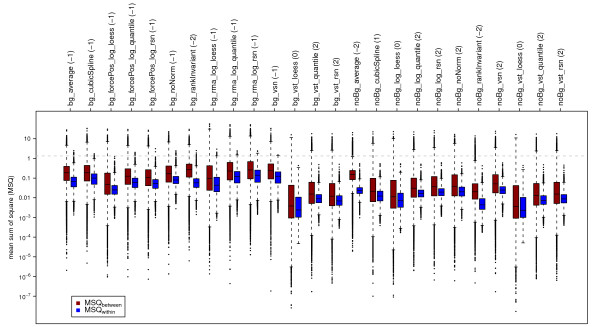
**Boxplots of MSQ_within _(blue) and MSQ_between _(red)**. MSQs were calculated based on the gene expression measured for the three sample groups analyzed, namely untreated HaCaT cells after 2, 4, and 12 hours. Results obtained for the different pre-processing methods used are displayed. The grey dashed line indicates the expected value for the MSQ_between _of 1.33 based on 6, 6, and 7 as measurements for the group means of four replicates for three time points.

Further indications for good normalization are the distributions of between (MSQ_between_) and within (MSQ_within_) group variances and their relation to each other. If genes are not differentially expressed, MSQ_between _should be comparable to MSQ_within_. For genes that are differentially expressed, MSQ_between _is supposed to be higher than MSQ_within_. Figure 4 displays the boxplots for MSQ_between _(red) and MSQ_within _(blue) values. Since we expect some genes to be differentially deregulated across the different time points under consideration, quantiles of MSQ_within _values should lie below the corresponding quantiles of the MSQ_between _values. For the differentially expressed genes, within group variance should be smaller than between group variance, whereas for the genes not differentially expressed, the respective MSQ_between _and MSQ_within _values should show no great difference. Small interquartile ranges (IQRs) of MSQ_within _are indicative for a comparable variability between genes.

To judge the values for MSQ, an MSQ_between _was calculated for artificial group means of log_2 _expression values for three time points based on four replicates. The group means used were (6, 6, 7) which resulted in an MSQ_between _of 1.33, indicated by a dashed grey line in Figure 4. The mean expression values of the artificial groups have been chosen such that they exhibit a log_2 _ratio of 1 when group 3 is compared to group 1 or group 2, reflecting a relevant difference between those groups. A good normalization method should result in similar expression values for replicates and thus in small MSQ_within _values hardly crossing this artificial MSQ_between_. Additionally, since we limited the whole data set to expressions measured for untreated HaCaT cells across time, we expect only few genes to be differentially expressed. Thus, only a few genes are assumed to result in an MSQ_between _above the artificial MSQ_between_.

Almost all boxplots representing MSQ_within _of background normalized data (bg_*) result in outliers crossing the artificial MSQ_between_, only those transformed using vst do stay below. Compared to other pre-processing methods, noBg_vst_loess, noBg_log_loess, and bg_vst_loess show a relatively wide IQR for both, MSQ_between _and MSQ_within_. Methods that meet the described behaviour by showing a low within group variability for which the quantiles generally exhibit lower values than the quantiles of the between group variabilities are bg_vst_quantile, bg_vst_rsn, noBg_log_quantile, noBg_log_rsn, noBg_noNorm, noBg_vsn, noBg_vst_quantile, and noBg_vst_rsn.

#### Density functions of MSQ_between _and MSQ_within_

Density functions of MSQ_between _and MSQ_within _should exhibit clear differences. This fact renders density functions of MSQ_between _and MSQ_within _as an additional option for investigating these values. Within group variability should be smaller than between group variability and most of the genes should show a between group variability similar to the within group variability, i.e. are not differentially expressed. Thus, the mode of MSQ_within _should be smaller than the mode of MSQ_between _and the peak of the function for MSQ_within _is supposed to be higher than the peak for MSQ_between_. Lean MSQ_within _functions, on the one hand, reflect a comparable within group variability for many genes. On the other hand, broader MSQ_between _functions indicate that at least some of the genes, i.e. the differentially expressed ones, show a higher between than within group variability. Ideal characteristics of density functions as described here are very similar to the characteristics of ideal boxplots mentioned in the previous section. In contrast to density functions, boxplots give a very rough idea about the distribution of the values, also depicting outliers. Density functions deliver a more detailed view of how the values are distributed across different ranges.

**Figure 5:**
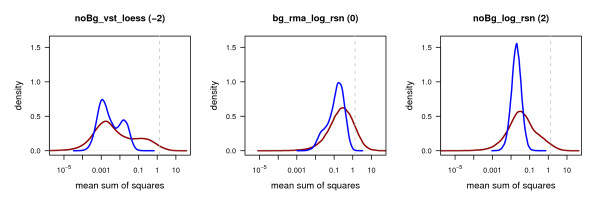
**Density plots of MSQ_within _(blue) and MSQ_between _(red)**. MSQs were calculated based on the gene expression measured for the three sample groups analyzed, namely untreated HaCaT cells after 2, 4, and 12 hours. The grey dashed line indicates the expected value for the MSQ_between _of 1.33 based on 6, 6, and 7 as measurements for the group means of four replicates for three time points. Three examples of different quality are shown. Based on the noBg_vst_loess pre-processing the MSQ_within _values show a strong bimodal distribution, for the bg_rma_log_rsn pre-processing the distribution is very asymmetric. The distributions for noBg_log_rsn based values reflect the desired behaviour. The quality values assigned are -2, 0, and 2, for noBg_vst_loess, bg_rma_log_rsn, and noBg_log_rsn, respectively. For an overview of the distributions for all pre-processing methods and their respective plots, see Additional file 2.

Figure 5 displays density functions of MSQ_between _(red) and MSQ_within _(blue) for three of the pre-processing methods, a complete overview is given in Additional file 2. In particular density plots representing the normalization methods noBg_log_quantile, noBg_log_rsn, and noBg_vsn best exhibit the desired behaviour. Unexpectedly the density functions of MSQ_within _generated by bg_vst_loess, noBG_log_loess, and noBg_vst_loess are bimodal. One reason for bimodal density functions could be a group of transcripts exhibiting higher variability compared to other transcripts. In general, it is expected that the data shows a consistent variability. Having the opportunity to choose between normalization methods resulting in unimodal or bimodal density functions for MSQ_within_, normalization methods leading to a unimodal distribution should be favoured.

A small overlap of the functions like for the values generated by the noBg_average normalization (Additional file 2) would indicate the unlikely event that most of the genes show a higher between than within group variability, i.e. are differentially expressed. Thus, this normalization method is probably not adequate.

#### Volcano plots

**Figure 6:**
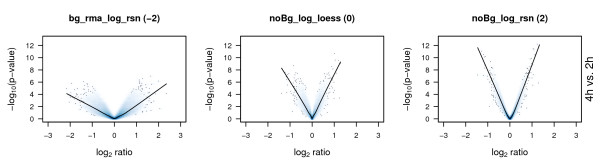
**Volcano plots**. Log_2 _ratios and p-values for the comparison of untreated HaCaT cells at 4 hours compared to 2 hours, 12 hours compared to 2 hours, and 12 hours compared to 4 hours were calculated based on the gene expression measured. Three examples of different qualities are displayed showing the -log_10_(p-value) against log_2 _ratio comparing 4 hours to 2 hours. The blue line represents a loess-curve fitted to the values. Quality values assigned to bg_rma_log_rsn, noBg_log_loess, and noBg_log_rsn are -2, 0, and 2, respectively. Pre-processing using bg_rma_log_rsn yields a very flat volcano like shape with p-values exhibiting a high degree of scattering, i.e. log_2 _ratios are overestimated and at the same time p-values are not very accurate. In contrast, noBg_log_loess better represents the expected range of log_2 _ratios (not many genes are assumed to heavily change their expression between different time points) but compared to noBg_log_rsn p-values still are not very accurate, i.e. show a high degree of scattering for equivalent log_2 _ratios. For a complete overview over all methods and all comparisons, see Additional file 3.

Volcano plots constitute a standard visualisation of microarray results. They are generated by plotting -log_10_(p-value) versus the respective log_2 _ratios. Due to the tendency of larger log_2 _ratios being connected to more significant -log_10_(p-values) a volcano like shape is generated. Pairwise comparisons (4 hours compared to 2 hours, 12 hours compared to 2 hours, and 12 hours compared to 4 hours) using a moderated t-statistic [21] were performed to calculate log_2 _ratios and p-values. Our aim is to detect normalization procedures yielding as correct estimates of log_2 _ratios as possible combined with as informative p-values as possible. As mentioned above higher log_2 _ratios should tend to have higher -log_10_(p-value). The loess fits of the log_2 _ratios and -log_10_(p-value) pairs (dark blue curves) of the volcano plots shown in Figure 6 and Additional file 3 shall neither be too flat nor too narrow and the scatter of the p-values for specific log_2 _ratios should not be too large.

All volcano plots based on rma background corrected data do not look very promising. The fitted curves are rather flat, i.e. even for high absolute log_2 _ratios the -log_10_(p-value) are relatively low. Additionally, the -log_10_(p-value) for similar log_2 _ratios tend to scatter extremely. Some volcanos, e.g. bg_average, bg_noNorm, bg_rankInvariant, and noBg_rankInvariant, show an unsymmetrical relation between p-values for negative and positive log_2 _ratios. Especially noBg_rankInvariant exhibits a bias towards small negative log_2 _ratios for which the respective -log_10_(p-value) seem to be relatively high. In this region the fitted curve shows a very steep, linear course. Volcano plots generated for all other methods are similar to what would be expected. Still they differ in the variance of the p-values and in that some of the fitted curves show a flatter shape than others. This reflects the fact that some normalization methods generate a smaller variance than others, resulting in lower fold changes but more significant p-values. Ultimately, a method with a reasonable trade off between fold change and variance has to be chosen and cut-off parameters for interesting genes have to be defined accordingly. Volcano plots best reflecting the desired properties in the context of our experiment were generated by noBg_log_quantile, noBg_log_rsn, and noBg_vsn. They show the least scattering of values around the fitted curves, but they probably underestimate fold changes. Plots produced bye bg_forcePos_log_loss, bg_forcePos_log_quantile, bg_forcePos_log_rsn, bg_vst_quantile, bg_vst_rsn, noBg_cubicSpline, noBg_log_loess, noBg_vst_loess, noBg_vst_quantile, and noBg_vst_rsn also fulfil the above mentioned criteria, but show more scattering.

#### Residual standard deviation against mean and minimum of gene expression levels

In an optimally normalized experiment the residual standard deviation of fitted gene expression intensities should be low and independent of the expression levels, i.e. the variance over the different expression levels should be stable. This is prerequisite for many statistical methods, like for example linear model fitting and moderate t-statistics [21], that are utilised for analysing gene expression data.

**Figure 7:**
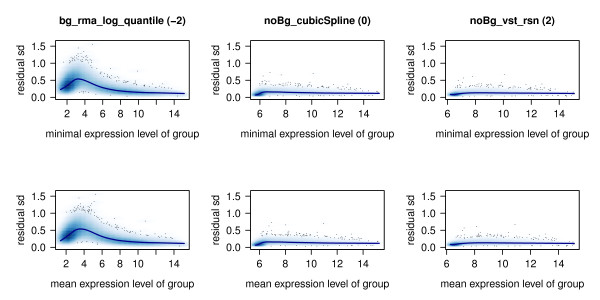
**Residual standard deviation against expression intensities**. Standard deviation of the residuals observed for the regression fitted to the expression intensities are plotted against minimum (upper row) and mean (lower row) expression intensity of each probe. The blue line represents a loess-curve fitted to the values. bg_rma_log_quantile exhibits very high deviation of residuals in ranges of lower expression intensities, whereas noBg_vst_rsn shows homogeneous and low deviations of residuals over the whole range of expression intensities. Compared to noBg_vst_rsn, residual standard deviations tend to be a bit higher and less homogeneous in small ranges of expression intensities when noBg_cubicSpline is used for pre-processing. Thus, scores of -2, 0, and 2 are assigned for bg_rma_log_quantile, noBg_cubicSpline, and noBg_vst_rsn, respectively. For an overview over all methods, see Additional files 4 and 5.

As displayed in Figure 7 and Additional files 4 and 5, all of the methods without background normalization (noBg_*) show a moderate or low variance in regions of no or hardly to measure expression. In contrast, nearly all of the background corrected methods (bg_*) result in high and, compared to the other methods, instable variance in the range of low intensity values. Extreme examples especially are rma background corrections and bg_vsn normalization procedures. An exception constitute methods using background normalization in conjunction with variance-stabilizing transformation (bg_vst_*) which in contrast to other procedures using background corrections perform especially well.

Methods which perform best with respect to variance stabilization across all expression levels are bg_vst_loess, bg_vst_quantile, bg_vst_rsn, noBg_vst_loess, noBg_vst_quantile, and noBg_vst_rsn.

#### Scatterplots of expression values

**Figure 8:**
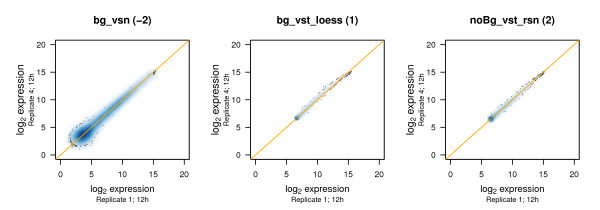
**Scatterplots between replicates**. After application of different normalization methods, expression values for the respective replicates at 12 hours are plotted against each other. bg_vsn as well as noBg_vst_rsn display a symmetrical distribution of expression values around the main diagonal (orange line), with bg_vsn exhibiting more scattering values especially obvious in low regions of expression. The scatterplot based on bg_vst_loess is slightly bended towards the upper diagonal based on a bias to higher values in the expression values for Replicate 4. Thus, scores of -2, 1, and 2 are assigned to the scatterplots based on bg_vsn, bg_vst_loess, and noBg_vst_rsn, respectively. For an overview over all methods, see Additional file 6.

Scatterplots are an easy and straightforward visualisation tool for judging the comparability of replicates. They clearly show whether high variances are to be expected and, if this is the case, in which range of the expression data. Figure 8 and Additional file 6 display the expression values of replicates plotted against each other. Our results by and large confirm previous findings. Some of the methods, for example bg_rma_log_loess, bg_rma_log_quantile, bg_rma_log_rsn, and bg_vsn, show high variance especially in the range of lower expression. Plots generated based on these procedures exhibit high variability between replicates. Some of the methods like for example bg_noNorm, bg_vst_loess, and noBg_average lead to asymmetric scatterplots indicating a bias in the expression values and a higher variability between replicates. Methods that perform well in stabilizing the variance across different expression levels, for example bg_vst_quantile, bg_vst_rsn, noBg_vst_loess, noBg_vst_quantile, and noBg_vst_rsn, could also be confirmed by the scatterplots. Additionally to those, noBg_cubicSpline and noBg_rankInvariant exhibit symmetric scatterplots with a very low degree of variance between replicates.

#### Pseudo-ROC curves

**Figure 9:**
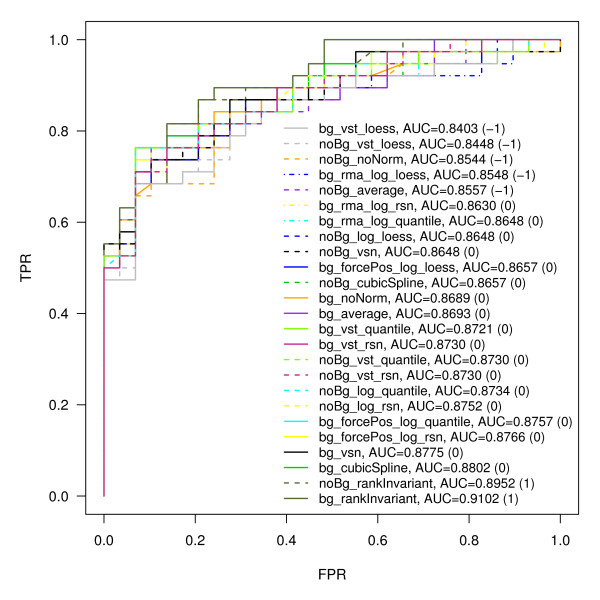
**Pseudo-ROC curves based on adjusted p-values**. Pseudo-ROC curves were calculated for the different pre-processing methods. FDR-adjusted p-values [35] of an F-statistic comparing the expression intensities measured for untreated and TGF-β stimulated HaCaT cells after 2 hours were used as a threshold. (TPR: true positive rate, FPR: false positive rate, AUC: area under curve).

In order to compensate for missing spike-in and dilution data a pseudo-ROC approach [22] mimicking the presence of true negatives has been conducted. The pseudo-ROC curve for each normalization method is a linear transformation of the true ROC curve. Common single number summaries used to score and compare ROC curves - the area under the curve (AUC) or the sensitivity at a given false positive rate - are area or distance based, and thus reduced by this transformation, but to the same degree for every curve. Aiming at the validation of normalization methods with respect to their ability to generate data exhibiting a good sensitivity to specificity ratio, expression intensities derived from TGF-β treated versus untreated cells at 2 h were compared. Based on the ROC curves' AUC (Figure 9, Additional file 7), all normalization methods perform relatively well in delivering values suited for separating true positives from true negatives. To assign quality values to the ROC curves, the AUC values were sorted and subsequently allocated to three bins of sizes 5, 18, and 2. Finally the bins were assigned quality values of -1, 0, and 1, respectively (Figure 1). bg_rankInvariant performs best with an AUC of 0.9102, whilst bg_vst_loess performs worst with an AUC of 0.8403.

### Analyses of bias based on qRT-PCR

qRT-PCR has been performed for mRNAs from eight genes that are known to be deregulated by TGF-β signalling to a varying degree (CDKN1A, CDKN2B, HAND1, JUNB, LINCR, RPTN, SERPINE1, and TSC22D1). By this means, it is possible to compare the results of the normalization methods to values that reflect the real abundance of the respective mRNA in the cells. Thus, we are able to evaluate the accuracy of the different pre-processing methods with respect to their bias. To guarantee that the comparisons of the normalization methods are not biased towards certain intensities, the mRNAs used in qRT-PCR experiments were chosen such that the respective signals on the chips cover a broad range of expression intensities (Additional file 8).

#### Correlation analysis of fold changes

**Figure 10:**
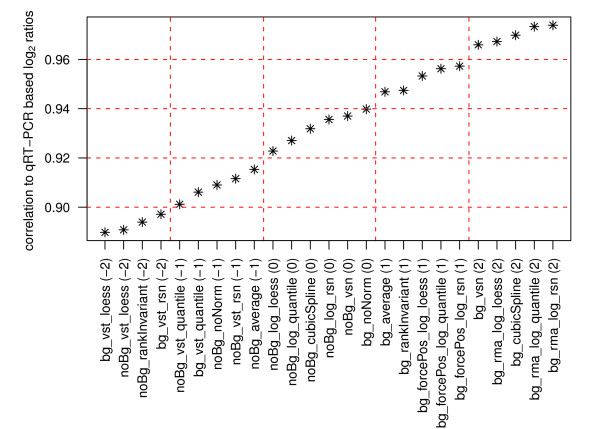
**Pearson correlation of log_2 _ratios for different normalization methods and qRT-PCR**. Correlations of log_2 _ratios were calculated for differently pre-processed gene expression data from BeadChip arrays and qRT-PCR based results. On the x-axis, pre-processing methods are ranked according to their correlation to qRT-PCR. The dashed red lines indicate the cut-offs used for assigning quality score between -2 (< 0.9) and 2 (>0.96).

Based on the different normalization procedures for the gene expression experiment and based on the qRT-PCR measurements (Additional file 8), Pearson correlations of the respective fold changes measured for TGF-β stimulated versus untreated cells at 2 hours, 4 hours, and 12 hours were calculated. Figure 10 displays the ranked correlation coefficients describing the relation between the different normalization methods and the TaqMan results. Quality values were assigned based on correlation cut-offs. A value of 2 is assigned to correlation coefficients ≥ 0.96, a value of 1 to coefficients between 0.94 and 0.96, a value of 0 to coefficients between 0.92 and 0.94, a value of -1 to coefficients between 0.9 and 0.92, and a value of -2 to correlation coefficients ≤ 0.9 (Figure 1). Values derived from most of the methods not utilising background correction (noBg_*) show a lower correlation to the TaqMan results than expression intensities that are background corrected (bg_*). An exception in this regard are methods that are based on vst transformation (bg_vst_*). These three methods are amongst the six methods resulting in the lowest correlation coefficient values. Correlation coefficients exhibiting high values are delivered by methods introducing BeadStudio's background correction combined with either rma background correction and log_2_-transformation (bg_rma_log_*), cubic spline normalization (bg_cubicSpline), or variance stabilizing normalization (bg_vsn).

#### Regression analysis

**Figure 11:**
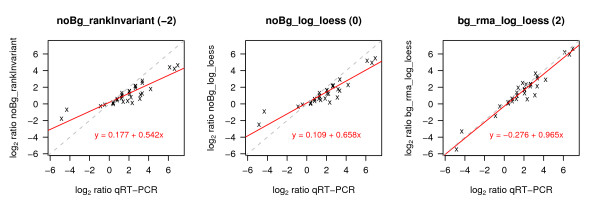
**Orthogonal regression between qRT-PCR and normalization based log_2 _ratios**. Regression of log_2 _ratios was conducted based on different normalization methods (y-axis) against qRT-PCR (x-axis). Equations and the respective regression lines are displayed in red. The grey dashed line indicates the main diagonal. Log_2 _ratios as calculated based on noBg_rankInvariant and noBg_log_loess pre-processing are overestimated in the lower and underestimated in higher ranges of log_2 _ratios. This over- and underestimation is more extreme for noBg_rankInvariant (intercept = 0.177, slope = 0.542) than for noBg_log_loess (intercept = 0.109, slope = 0.658). Data pre-processed using bg_rma_log_loess hardly over- or underestimates the data (intercept = -0.276, slope = 0.965). This results in scores of -2, 0, and 2 for noBg_rankInvariant, noBg_log_loess, and bg_rma_log_loess, respectively. An overview over the results for all pre-processing methods is given in Additional file 9.

**Figure 12:**
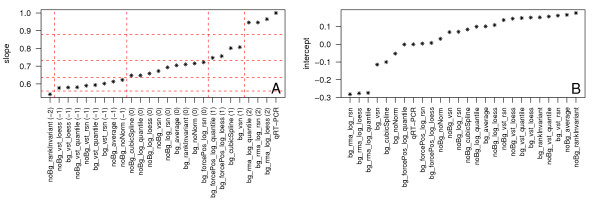
**Results of orthogonal regression**. Ranking of slope (A) and intercept (B) of the orthogonal regression lines as displayed in Figure 11). and Additional file 9. For the slope, quality scores are assigned from -2 to 2 based on the jumps visible and indicated by the red dashed lines.

To investigate the linear relationship between fold changes as determined by TaqMan and gene expression data, a linear regression analysis was performed by minimizing the sum of squares of the Euclidean distance of points to the fitted line ('orthogonal regression', Figure 11, Additional file 9). This method was chosen because there is no clear assignment of dependent and independent variables. Figure 12 displays the ranking of the different methods according to the slopes of the orthogonal regressions. Following rules apply for results of these analyses: The closer the slope is to 1, the better the respective normalization method reflects the qRT-PCR results in a linear manner. In this situation the deviation of the intercept from 0 indicates a constant under- or overestimation of the change of mRNA abundance across the whole range of fold changes. An intercept < 0 stands for an underestimation and an intercept > 0 for an overestimation of fold changes. In the case that the slope deviates from 1 the difference between qRT-PCR based fold changes and normalized expression based fold changes depends on the size of the fold change. Here, on the one hand, an intercept near 0 implies a continuous over- (slope > 1) or underestimation (slope < 1). Depending on the slope, an intercept deviating from 0, on the other hand, indicates overestimation for a certain range of values and underestimation for another range of values. Regardless of the intercept, the most important point in our case is that the scatterplots are generally linear, with low variability and a slope close to 1. In accordance to previous results, all expression values that are transformed using vst together with noBG_rankInvariant result in slopes that exhibit the largest deviation from 1. Fold changes calculated based on rma background correction and log_2_-transformation (bg_rma_log_*) best fit the qRT-PCR results (Figure 12).

## Conclusions

It is important to select appropriate pre-processing methods for a given data set based on the experimental setup used. On the one hand, if sample sizes of the different groups are relatively small, it is crucial to achieve a homogeneous variance for the groups. On the other hand, if sample sizes are large, variances can be estimated separately and one should focus on unbiased fold changes. Since the sample sizes for the current data set are rather small (three to four replicates per group), a stable variance is more important than an exact representation of the fold change. In general, the data should be normalized without too much reducing real variations. Figure 1 summarizes the quality measures for all methods we investigated, demonstrating the background for the final choice. Clustering of the quality scores assigned reveals two major tendencies based on background normalization. On the one hand, data that was background normalized (bg_*) tend to better reflect the real fold changes, i.e. show less bias. On the other hand, pre-processing without background normalization (noBg_*) leads to a more homogeneous variance. Accurately defined, constant experimental conditions across all experiments as well as their parallel conduction probably have lead to a relatively consistent background level across all samples. Since background correction could introduce additional variation, these could be the reasons why, for our data set, data that was not background normalized (noBg_*) in general provides better stabilization of variance than background normalized data (bg_*). Methods combining background normalization with vst (bg_vst_*) constitute an exception. Here, vst leads to a better stabilization of variance while introducing more bias. As vst estimates an offset for the background based on the data [9], noBg_vst_* and bg_vst_* pre-processing methods could lead to similar results.

One has to keep in mind that, based on the individual analyses, there are several methods resulting in nearly equal quality. Therefore, it is not possible to give a well-defined rationale for using only one specific method. After excluding the methods that clearly violate the imposed criteria, the decision is still subjective. It, for example, depends on whether one would like to account for a good estimate of fold changes or a small and homogeneous variance. Finally the decision remains based on experience; yet, with the analyses and criteria described here, we provide a recommendation on how to pre-select appropriate methods. Since, for our data set, we intended to achieve a low and homogeneous variance, we provided more and to a certain degree overlapping statistics investigating variance. In case the focus is on a good estimate of the fold change, the researcher should higher account for statistics investigating this measure. Correlation to qRT-PCR or slope and intercept of the regression between qRT-PCR and gene expression fold changes are examples of analyses that could be of higher interest in this context. Focusing on variance, best suited for the data set analysed here are noBg_log_quantile and noBg_log_rsn. Although log_2_-transformation in combination with quantile normalization has been approved as performing relatively well by Du *et al*. [17] and Dunning *et al*. [8,10], we decided to make use of robust spline normalization (rsn). In addition to our measures it was selected because rsn is aiming at combining the positive effects of quantile normalization, i.e. preservation of the rank order, and spline interpolation, i.e. continuous mapping of the values, but at the same time circumventing their drawbacks, i.e. discontinuous mapping of intensity values and no rank preservation, respectively [17,23]. Surprisingly, the use of vst as recommended by Dunning *et al*. [10] and by Du *et al*.[9,17,23] and the combination of vst with rsn as successfully used by Du *et al*. [23] did not perform as well as expected. Reasons for this could be the different experimental setups (two replicates per group in the Barnes setup [24] used for validation of vst compared to three to four replicates in our setup) or the use of a newer Illumina chip technology, namely HumanHT-12 v3 chips, in our experiment. vst has been validated based on a pre-released version of the HumanRef-8 v1 Expression BeadChip that contained 19 (25% quantile) to 30 (75% quantile) beads per probe. On the HumanHT-12 v3 chips an average of only 15 beads per probe is available. Since vst makes use of those technical replicates, this could lead to a slightly worse performance on the new chip generation. In general, vst still performs well in stabilizing the variance but is outperformed by noBg_log_quantile, noBg_log_rsn, and noBg_vsn in reflecting the results measured by qRT-PCR. When utilising BeadStudio normalizations, in accordance with Dunning *et al*. [8,10] who advised against the use of background normalization, we recommend using cubic spline without background normalization (noBg_cubicSpline). As displayed in Figure 1, noBg_cubicSpline outperforms all other BeadStudio normalization methods.

Spike-in or dilution data is frequently used for evaluating different normalization methods [5,7-10]. If no such data is available for the microarray chip type used, we propose to perform qRT-PCR for genes covering different spectra of expression intensities in order to obtain a measure for judging the quality of pre-processing methods. Thereby, it becomes possible to get an idea of how well different normalization methods are able to reflect the real changes in expression intensities across different expression levels.

In summary, we provide statistical measures based on which researchers can decide on the best suited pre-processing scenario for their own experimental design. If no spike-in data is available, we recommend conducting qRT-PCR for selected, representative transcripts. Thereby, it is possible to estimate the bias of log_2 _ratios obtained from normalized data. In conjunction with the measures for the variability of the data finally the basis for weighing well measured changes versus low and homogeneous variance is delivered and by this means selecting an appropriate normalization method is possible.

## Methods

### Biological experiments

#### Cell culture

HaCaT cells were cultured under standard conditions (REF). Cells were seeded in 96-well (ELISA) or in 24-well (RNA expression profiling) plates and grown overnight to a confluence of approximately 70%. Cells were starved for 3 hours in DMEM containing no FCS and subsequently stimulated with 5 ng/ml of TGF-β1 (R&D Systems) or left unstimulated as controls for 2, 4, and 12 hours.

#### RNA extraction

RNA isolation was carried out using a MagMAX™ Express-96 Magnetic Particle Processor and the MagMAX™-96 Total RNA Isolation Kit according to the manufacturer's protocol. Total RNA concentration was quantified by fluorescence measurement using SYBR Green II (Invitrogen) and a Synergy HT reader (BioTek) as previously described [25]. The RNA quality was characterized by the quotient of the 28 S to 18 S ribosomal RNA electropherogram peak using an Agilent 2100 bioanalyzer and the RNA Nano Chip (Agilent).

#### Amplification, labeling and BeadChip hybridization of RNA samples

Illumina TotalPrep RNA Amplification Kit (Ambion) was used to transcribe 200 ng toRNA according to the manufacture's recommendation. A total of 700 ng of cRNA was hybridized at 58°C for 16 hours to the Illumina HumanHT-12 v3 Expression BeadChips (Illumina). BeadChips were scanned using an Illumina BeadArray Reader and the Bead Scan Software (Illumina). Data is publicly available in ArrayExpress [26] (E-MTAB-265).

#### qRT-PCR

Quantitative Real-Time Polymerase Chain Reaction (qRT-PCR) was conducted for eight genes (CDKN1A, CDKN2B, HAND1, JUNB, LINCR, RPTN, SERPINE1, and TSC22D1) known to be deregulated at at least one time point by TGF-β stimulation.

mRNA expression levels of the eight genes were determined by qRT-PCR analysis using a 7900HT Fast Real-Time PCR System (Applied Biosystems) and the Universal ProbeLibrary System (Roche). Gene specific forward and reverse primer sequences were designed using the Universal Probe Library Assay Design Center (Roche). Total RNA was transcribed into cDNA using the High Capacity cDNA Reverse Transcription Kit (Applied Biosystems) according to the manufacture's instructions. qRT-PCR is carried out in a final volume of 12 μl in three replicates for each cDNA sample. Levels of RNA polymerase II were used for normalization of the data. ΔΔCT method was used to relatively quantify mRNA levels of treated samples compared to untreated controls (Additional file 8). Data is publicly available in ArrayExpress [26] (E-MTAB-265).

### Data processing

Data has been processed with BeadStudio version 3.0 and the R Language and Environment for Statistical Computing (R) 2.7.0 [27,28] in combination with Bioconductor 2.2 [29]. The Bioconductor lumi package [17] has been used for quality control. 25 combinations of background correction, transformation, and normalization methods displayed in Figure 1 were calculated either with methods from BeadStudio, with methods available in the lumi package, or with a combination of BeadStudio and lumi methods.

#### BeadStudio pre-processing

The normalizations executed by Illumina BeadStudio were all applied to the expression values on the original scale. If background adjustment was performed, we used the standard background normalization offered by BeadStudio (bg_*). Cubic Spline, Rank Invariant, and Average methods were used for normalization (for details see BeadStudio Gene Expression Module User Guide [30]). Expression values were then log_2_-transformed.

#### R pre-processing

*_noNorm data has been log_2_-transformed using the lumiT() function. Thus, for background corrected data forcePos has automatically been conducted.

forcePositive (forcePos) [17] or rma background adjustment (bgAdjust.affy) [4] available through the lumiB() function of the lumi package were used to transform negative values which can result from BeadStudio background normalization to positive scale to be able to log_2_-transform the expression values. Background correction referred to as noBg implies that the background normalization has not been applied.

For transforming the data, a simple log_2_-tranformation (log) or variance-stabilizing transformation (vst) [9] was used.

Data was normalized using quantile normalization (quantile) [31], robust spline normalization (rsn) [17], local regression (loess) [32], or variance stabilization and normalization (vsn) [18]. vst as well as vsn can handle negative values in the data. Thus, neither forcePos nor rma was applied as pre-processing for any of those two methods to not unnecessarily modify the values in artificial ways.

All methods used are implemented in the R packages affy [33], vsn [18], or lumi [17].

### Statistical measures

In the following, the statistical measures used are briefly summarized. Unless otherwise noted, all statistical calculations were performed using R. A small R-package to conduct and reproduce the described analyses is available from the authors upon request. For the visualizations displayed in Figures 3, 6, 7, 8, and Additional files 2, 4, 5, 6, and 7 the smoothScatter() function as implemented within the Bioconductor package geneplotter [34] has been used.

#### Signal to noise ratios

One aim of normalization is to minimize, for each gene, the within group variability while maximizing the between group variability also referred to as mean sum of square within

and mean sum of square between

respectively.

Here, k represents, for a given gene, the number of groups, n_i _the size of group i,  the mean expression level of group i, the total mean, N the total number of observations, and x_ij _the j^th ^value in group i. The aim is to maximize  which follows an F-statistic with (k -1; N-k) degrees of freedom. The results for this test are displayed in Figures 2 to 5. For artificial group means  = 6,  = 6, and  = 7, k = 3, n_1 _= n_2 _= n_3 _= 4, and  results to 1.33 and is indicated in Figures 4 and 5 by a grey dashed line. The FDR-corrected [35] p-values for the F-statistic were summarized using their empirical distribution function (Figure 2).

#### Pseudo-ROC curves

One of the main uses of expression arrays is the identification of genes that are differentially expressed under various experimental conditions. A typical identification rule filters genes with p-values and/or fold change exceeding a given threshold. Given a set of known true positives (TP) and false positives (FP), Receiver Operator Characteristic (ROC) curves offer a graphical representation of both specificity and sensitivity for such a detection rule. ROC curves are created by plotting the true positive rate (sensitivity) against false positive rate (1-specificity) obtained at each possible threshold value. Since we only know about TP (20 genes known to be deregulated by TGF-β), we made use of so-called pseudo-ROC curves [22]. The TNs were randomly sampled from the set of transcripts remaining when subtracting the TPs from all transcripts. As a threshold, we used FDR-adjusted p-values [35] of an F-statistic as previously described, this time based on the sample groups for untreated and TGF-β stimulated HaCaT cells at 2 hours.

#### Log_2 _ratios, residual standard deviation, and p-values

The log_2 _ratios, residual standard deviation, and p-values were calculated using linear models in combination with the moderated t-statistic as supplied by limma [21].

#### Regression analysis of fold change values and qRT-PCR measurements

To get an overall impression of how good of a fit of the fold change levels detected using the different normalization methods to the qRT-PCR results are, an orthogonal regression for the observations was performed using the princomp() function as available in the basic R environment.

## Naming conventions

The following naming conventions are used to refer to different normalization methods:

### R normalizations

<background correction>_<transformation>_<normalization>, where:

•    <background correction>={bg_forcePos, bg_rma, noBg},

•    <transformation>={log, vst},

•    <normalization>={loess, quantile, rsn}, and

•    <transformation>_<normalization>={vsn}.

### BeadStudio normalizations

<background normalization>_<normalization>, where

•    <background normalization>={bg, noBg} and

•    <normalization>={cubicSpline, rankInvariant, average}.

## Authors' contributions

PB did the laboratory work, KF-C and RS conducted the statistical and bioinformatics analyses, CI, WH, BB and RE supported the statistical analyses, CI supervised the statistical analyses, DM and KQ supervised the project. RS and PB wrote the manuscript and all authors proofread the manuscript. All authors read and approved the final manuscript.

## Supplementary Material

Additional file 1 **-log_10_(p-values) against MSQ_between _where MSQ_between _≤ 5**. MSQs were calculated based on the gene expression measured for the three sample groups analyzed, namely untreated HaCaT cells after 2, 4, and 12 hours. Results obtained for the different pre-processing methods used are displayed. The blue line represents a loess-curve fitted to the values. Click here for file

Additional file 2 **Density plots of MSQ_within _(blue) and MSQ_between _(red)**. MSQs were calculated based on the gene expression measured for the three sample groups analyzed, namely untreated HaCaT cells after 2, 4, and 12 hours. Results obtained for the different pre-processing methods used are displayed. The grey dashed line indicates the expected value for the MSQ_between _of 1.33 based on 6, 6, and 7 as measurements for the group means of four replicates for three time points. Click here for file

Additional file 3 **Volcano plots**. Log_2 _ratios and p-values for the comparison of untreated HaCaT cells at 4 hours compared to 2 hours, 12 hours compared to 2 hours, and 12 hours compared to 4 hours were calculated based on the gene expression measured. Displayed are the -log_10_(p-value) against log_2 _ratio for the respective comparisons and the different normalization methods used. The blue line represents a loess-curve fitted to the values. Click here for file

Additional file 4 **Residual standard deviation against minimum expression intensity**. For each pre-processing method, standard deviation of the residuals observed for the regression fitted to the expression intensities are plotted against minimum expression intensity of each probe. The blue line represents a loess-curve fitted to the values. Click here for file

Additional file 5 **Residual standard deviation against mean expression intensity**. For each pre-processing method, standard deviation of the residuals observed for the regression fitted to the expression intensities are plotted against mean expression intensity of each probe. The blue line represents a loess-curve fitted to the values. Click here for file

Additional file 6 **Scatterplots between replicates**. After application of different normalization methods, expression values for the replicates are plotted against each other. The orange line indicates the main diagonal. Click here for file

Additional file 7 **Ranking of AUC values**. AUC values as calculated for the pseudo-ROC analysis displayed in Figure 9 are ranked and cut-offs for the three bins are chosen based on the jumps visible at 0.86 and 0.89. Click here for file

Additional file 8 **Results of qRT-PCR**. 2^-ΔΔCt ^[37] values represent the observed fold changes between HaCaT cells stimulated with TGF-β (UT+TGFβ) and unstimulated cells (UT) at the three different time points measured. Click here for file

Additional file 9 **Orthogonal regression between qRT-PCR and normalization based log_2 _ratios**. Regression of log_2 _ratios based on different normalization methods (y-axis) against qRT-PCR log_2 _ratios (x-axis). Equations and the respective regression lines are displayed in red. The grey dashed line indicates the main diagonal. Click here for file
