# Supplementary figures and images for: Comparison of normalization methods for Illumina BeadChip HumanHT-12 v3
Source: BMC Genomics. 2010 Jun 2;11:349. doi: 10.1186/1471-2164-11-349 (PMC3091625; doi:10.1186/1471-2164-11-349)

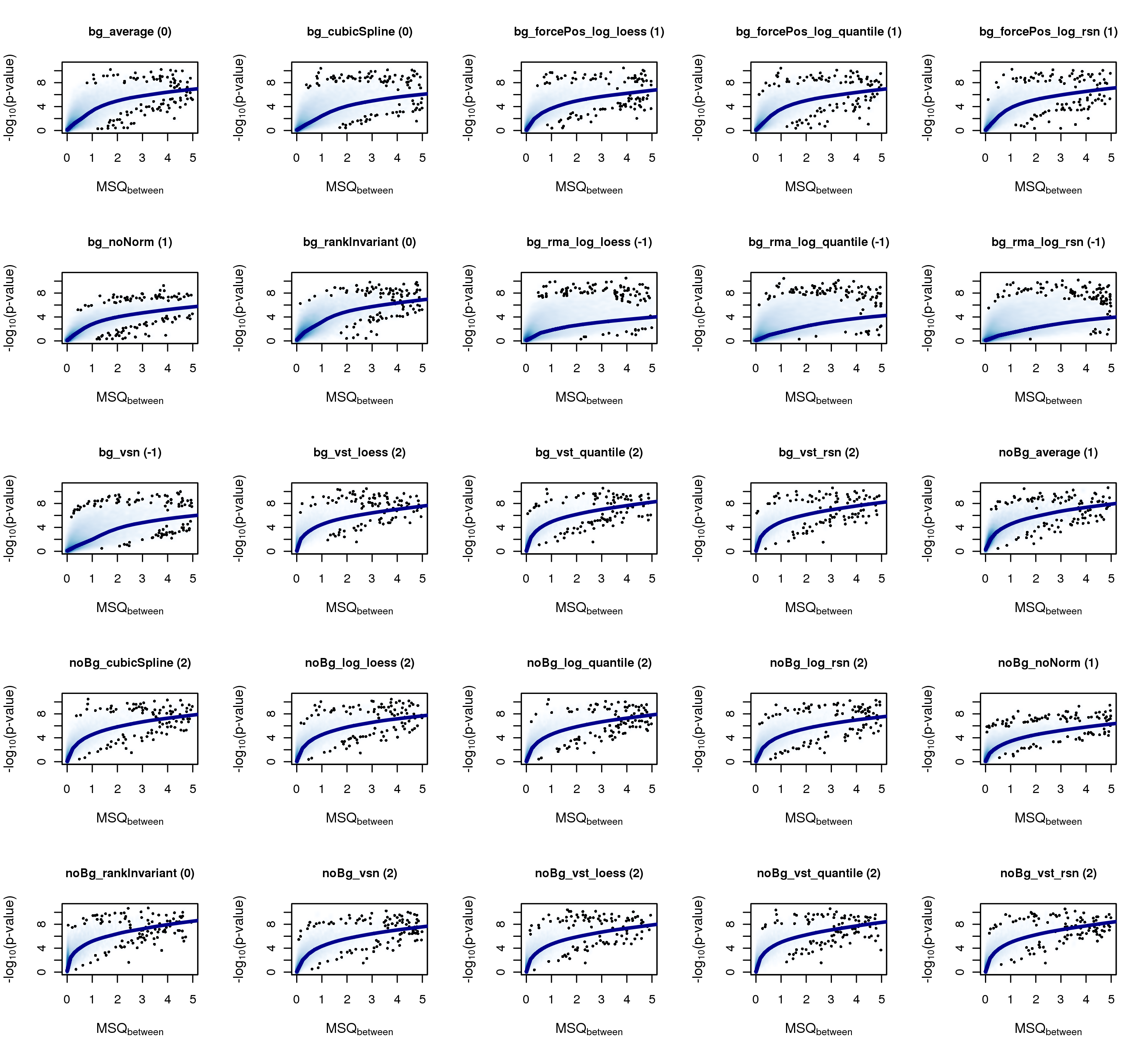

Supplement: Additional file 1 — -log10(p-values) against MSQbetween where MSQbetween ≤ 5. MSQs were calculated based on the gene expression measured for the three sample groups analyzed, namely untreated HaCaT cells after 2, 4, and 12 hours. Results obtained for the different pre-processing methods used are displayed. The blue line represents a loess-curve fitted to the values. [file 1471-2164-11-349-S1.PNG]

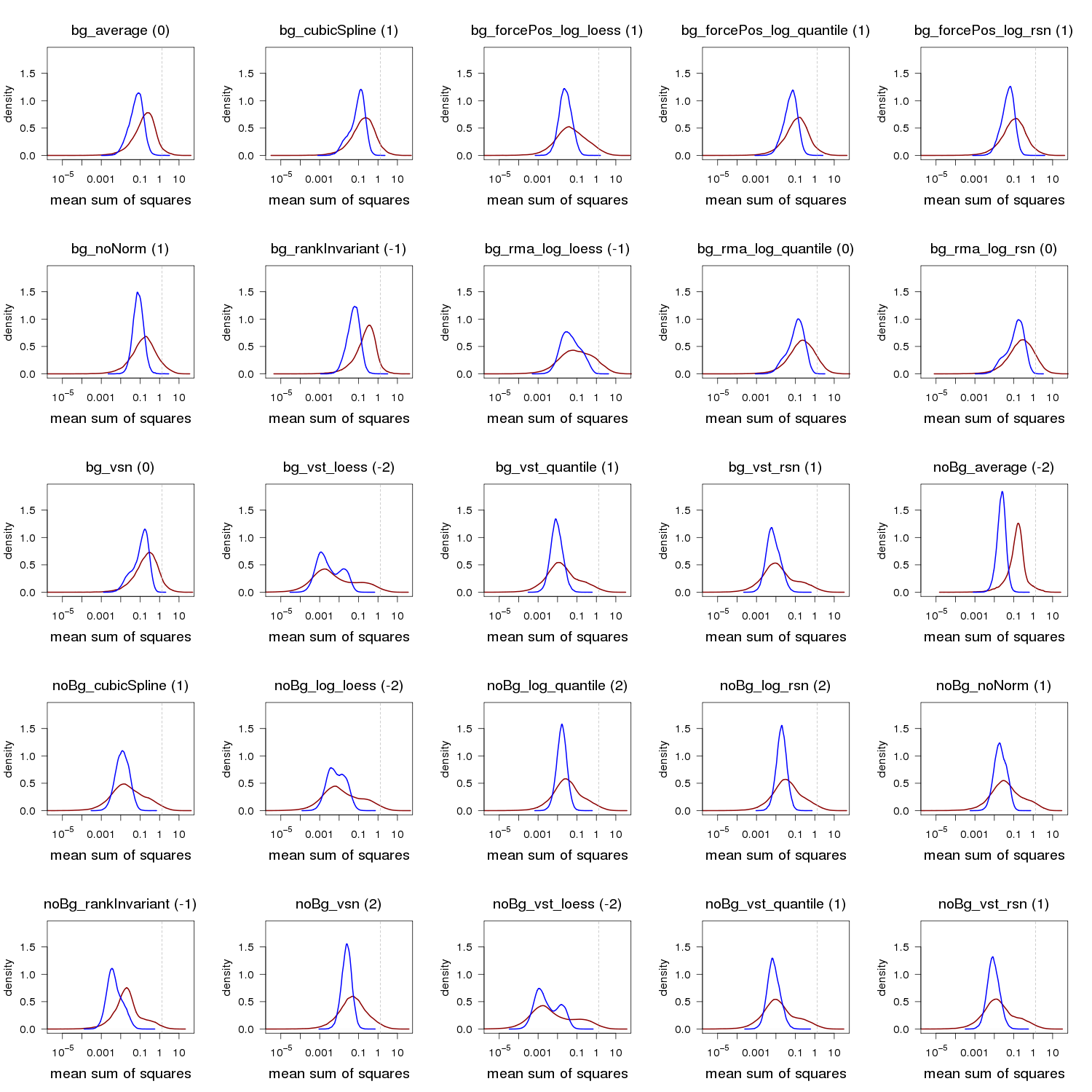

Supplement: Additional file 2 — Density plots of MSQwithin (blue) and MSQbetween (red). MSQs were calculated based on the gene expression measured for the three sample groups analyzed, namely untreated HaCaT cells after 2, 4, and 12 hours. Results obtained for the different pre-processing methods used are displayed. The grey dashed line indicates the expected value for the MSQbetween of 1.33 based on 6, 6, and 7 as measurements for the group means of four replicates for three time points. [file 1471-2164-11-349-S2.PNG]

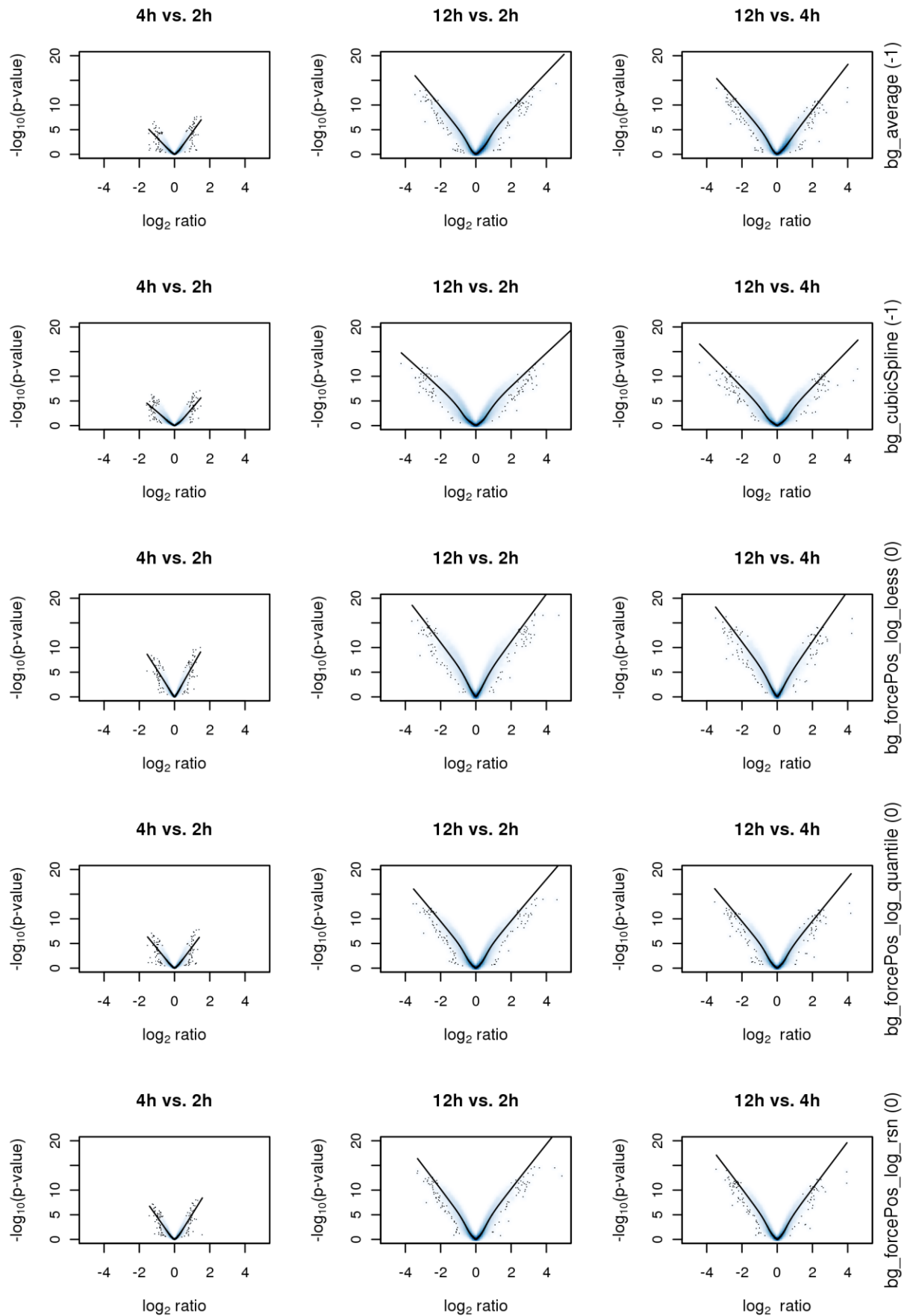

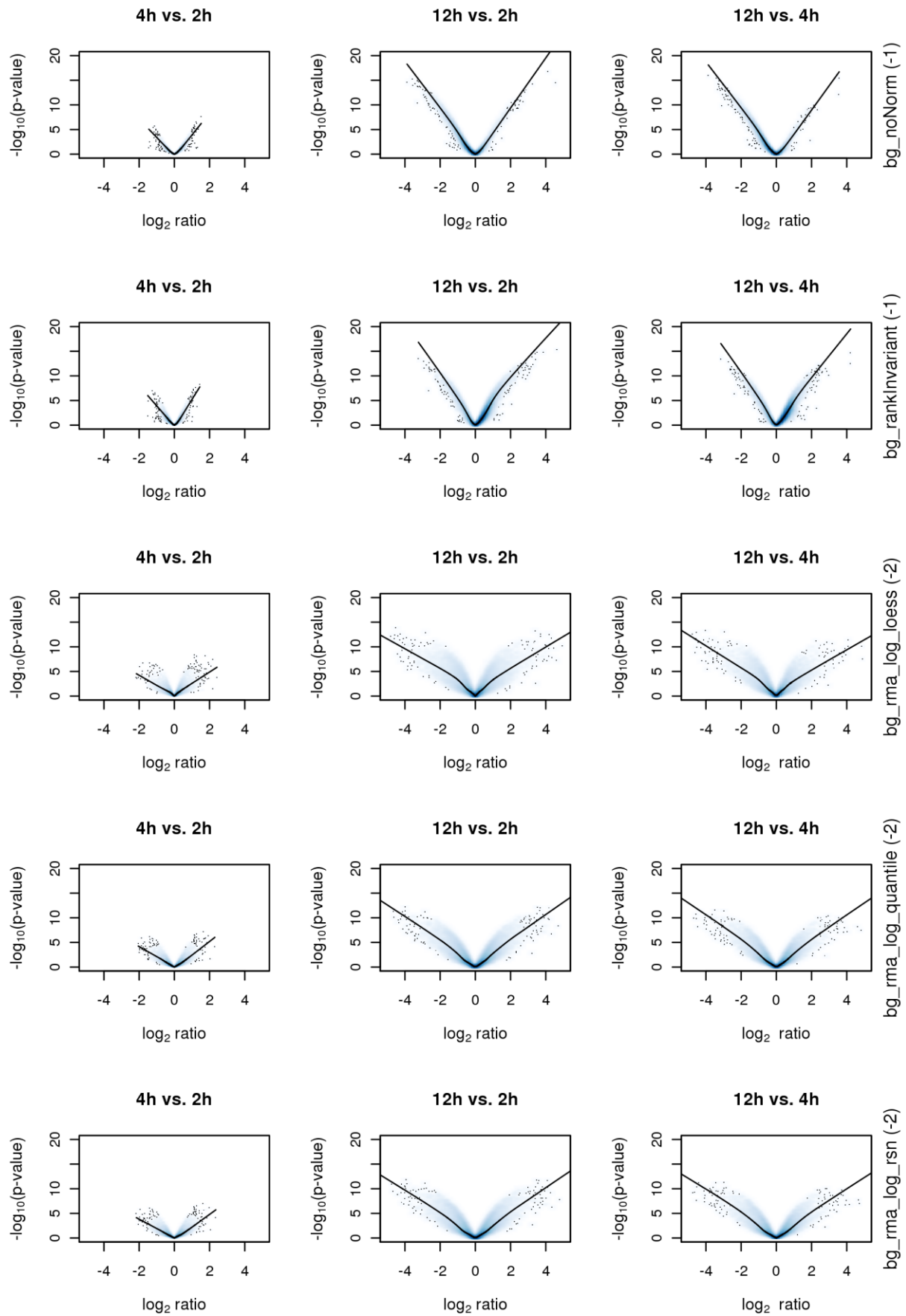

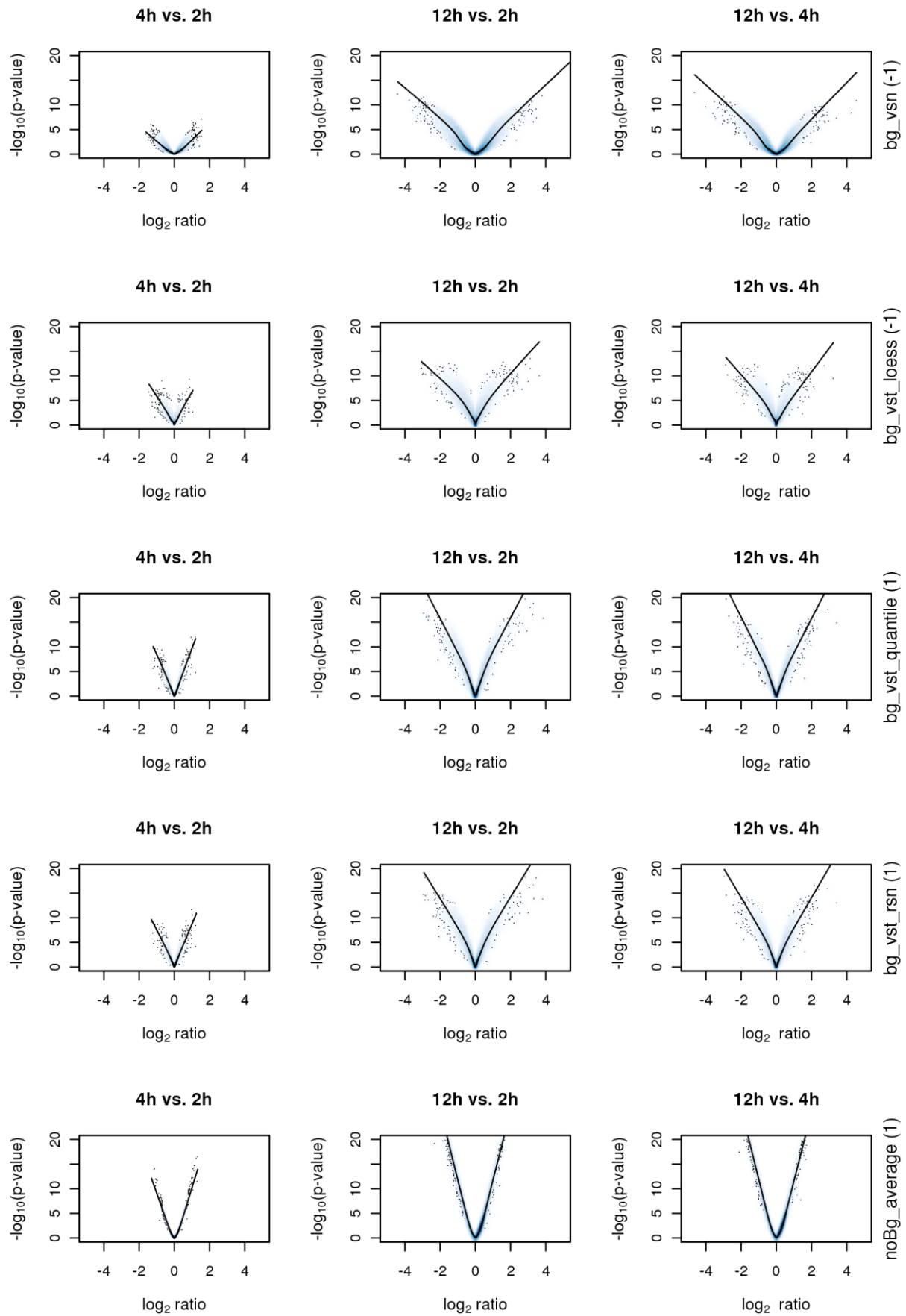

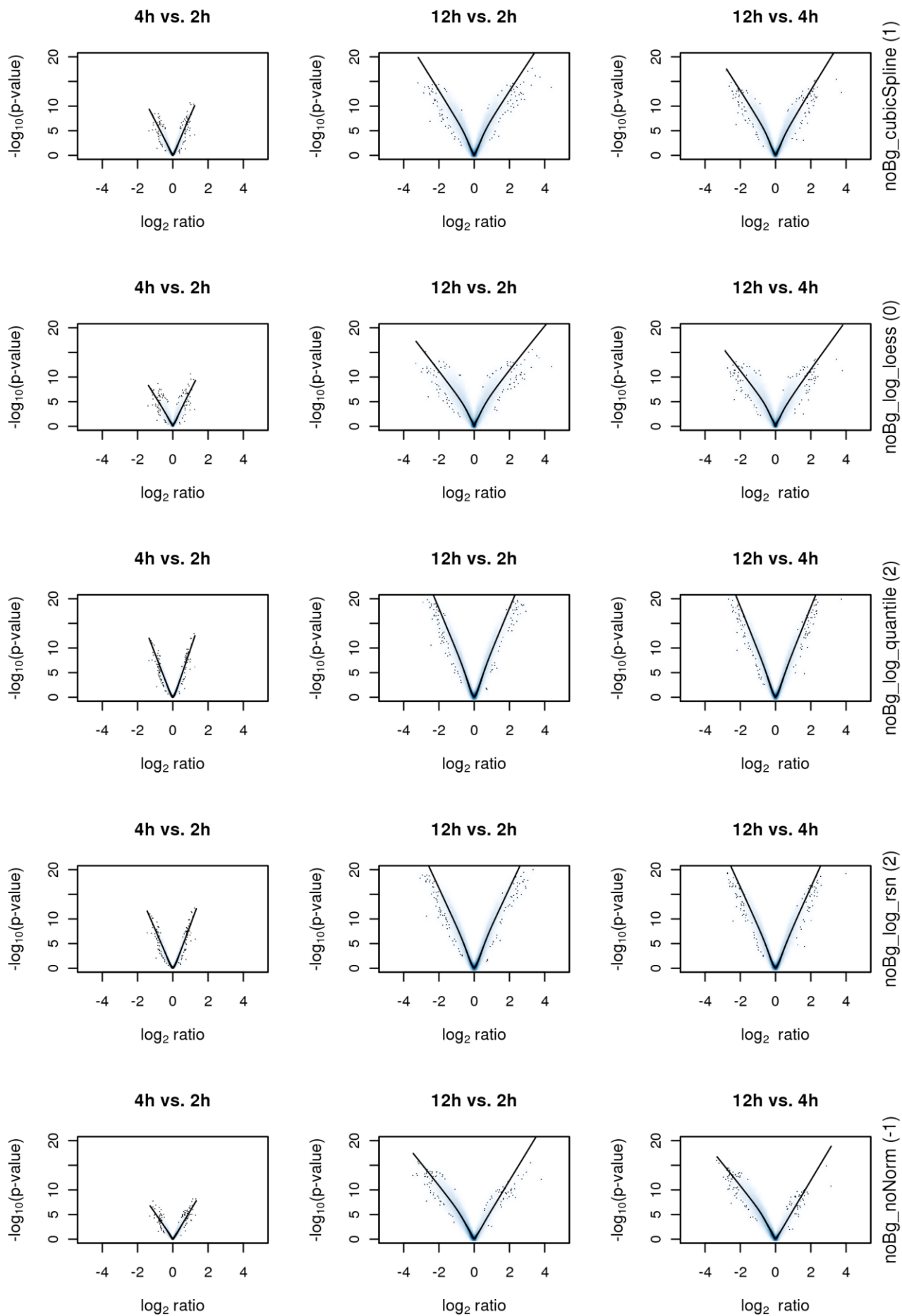

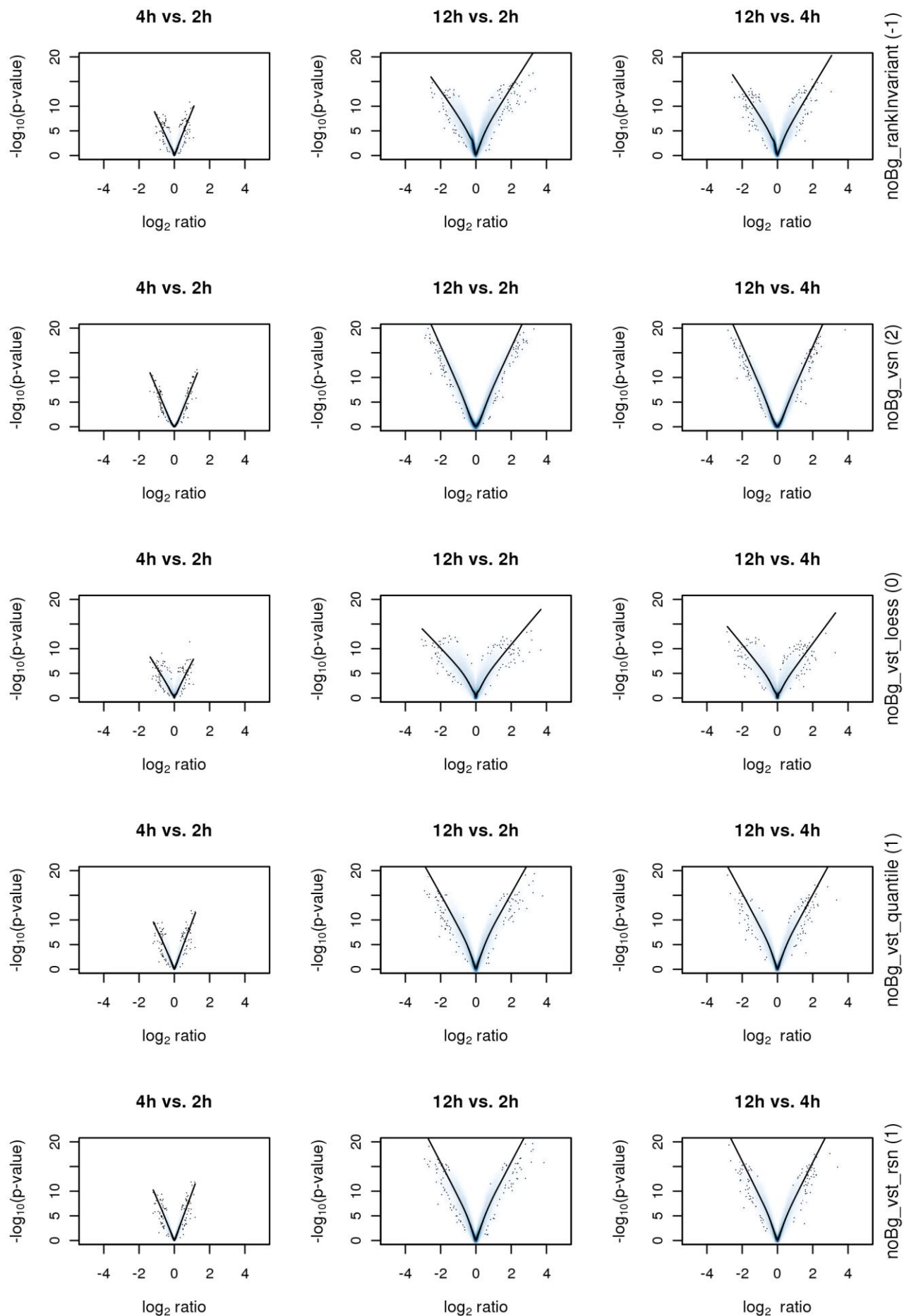

Supplement: Additional file 3 — Volcano plots. Log2 ratios and p-values for the comparison of untreated HaCaT cells at 4 hours compared to 2 hours, 12 hours compared to 2 hours, and 12 hours compared to 4 hours were calculated based on the gene expression measured. Displayed are the -log10(p-value) against log2 ratio for the respective comparisons and the different normalization methods used. The blue line represents a loess-curve fitted to the values. [file 1471-2164-11-349-S3.PDF]

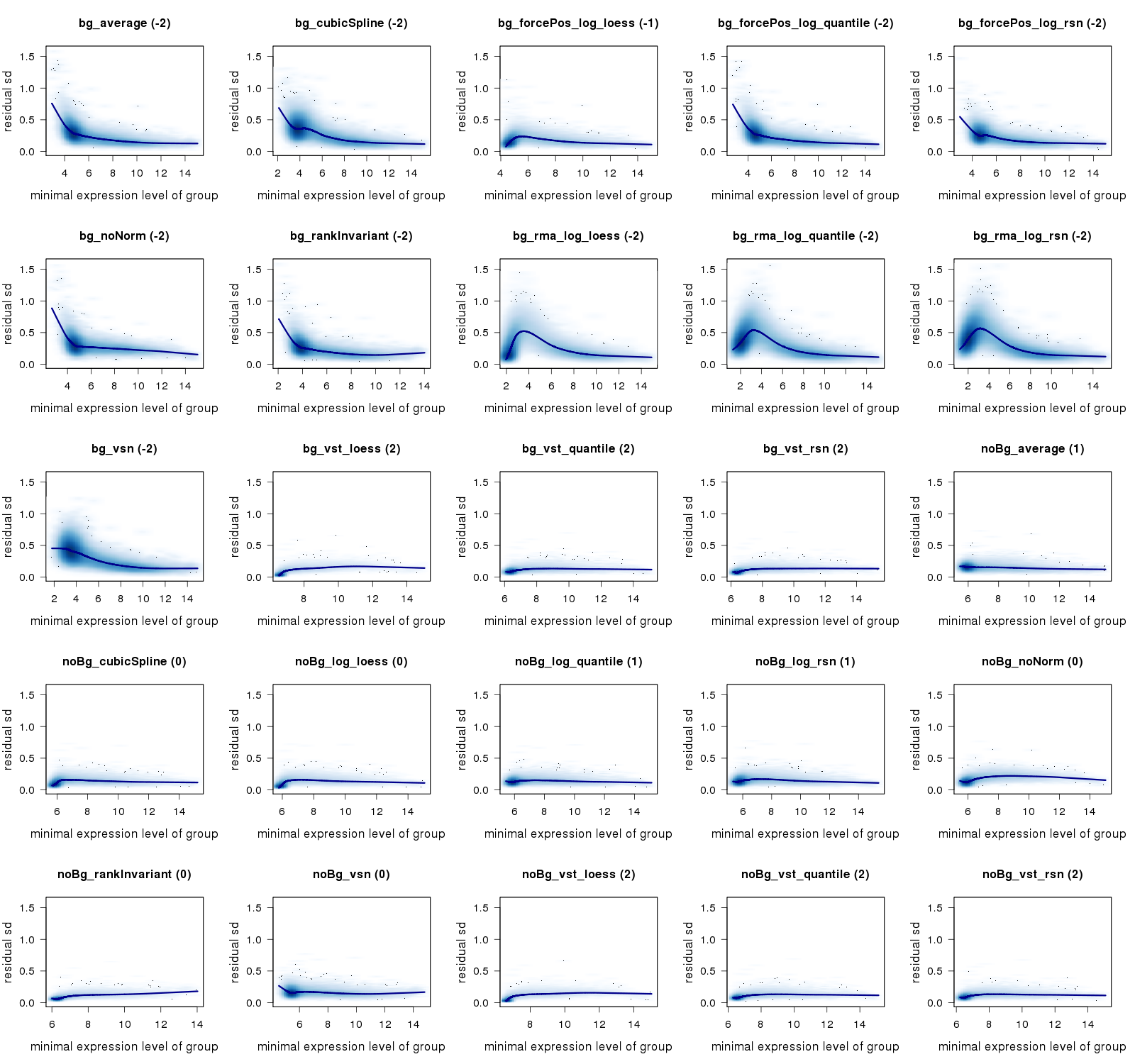

Supplement: Additional file 4 — Residual standard deviation against minimum expression intensity. For each pre-processing method, standard deviation of the residuals observed for the regression fitted to the expression intensities are plotted against minimum expression intensity of each probe. The blue line represents a loess-curve fitted to the values. [file 1471-2164-11-349-S4.PNG]

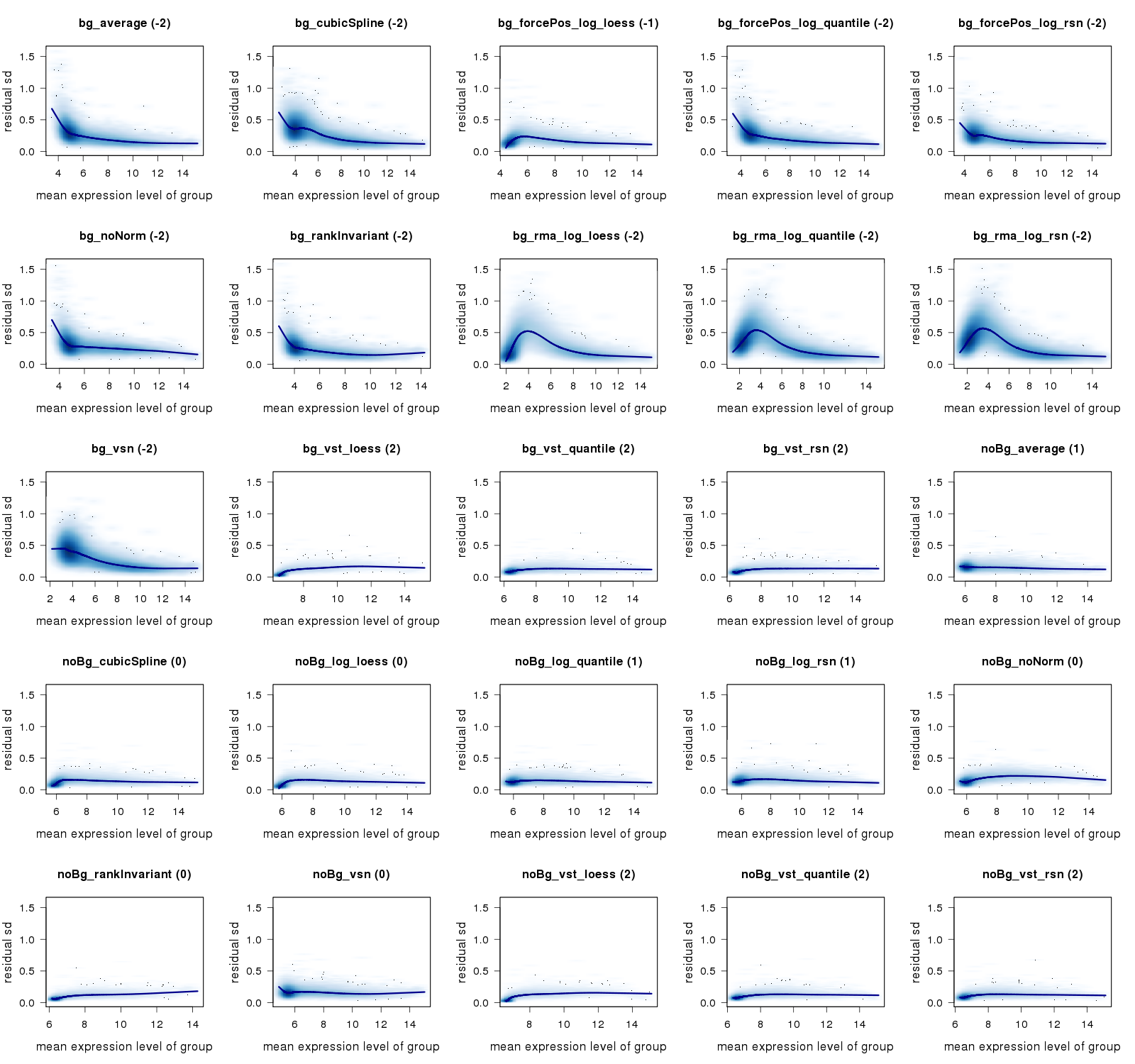

Supplement: Additional file 5 — Residual standard deviation against mean expression intensity. For each pre-processing method, standard deviation of the residuals observed for the regression fitted to the expression intensities are plotted against mean expression intensity of each probe. The blue line represents a loess-curve fitted to the values. [file 1471-2164-11-349-S5.PNG]

AUC

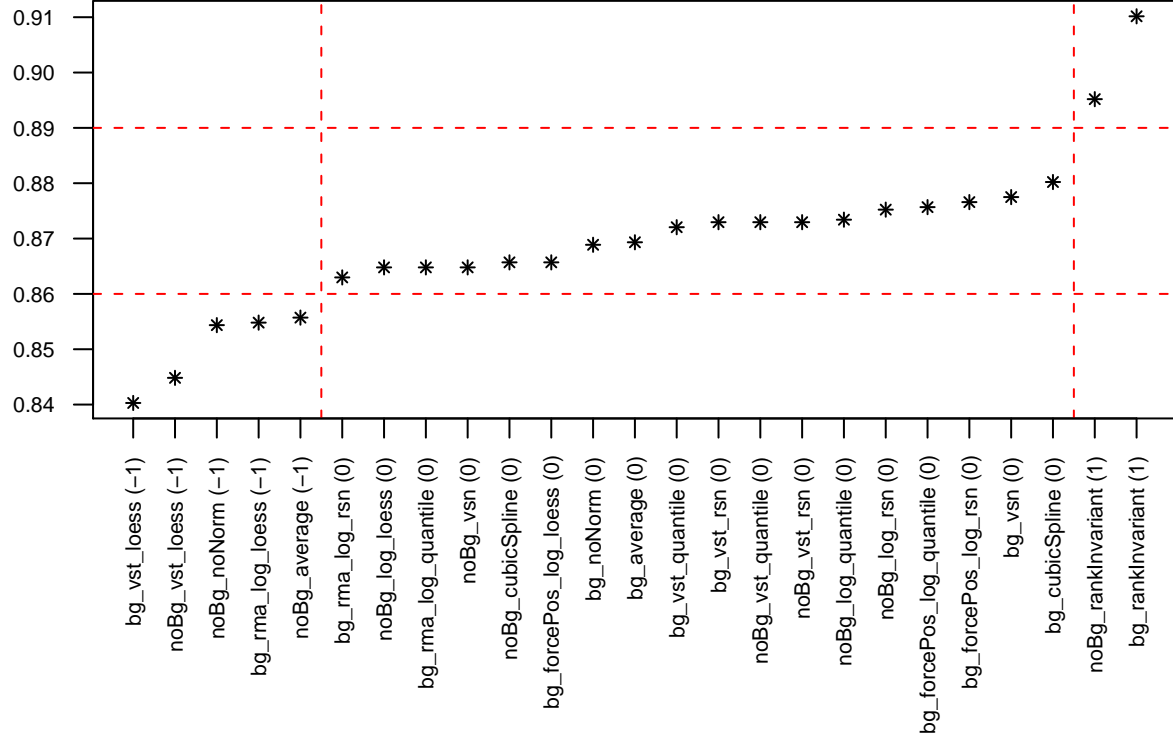

Supplement: Additional file 7 — Ranking of AUC values. AUC values as calculated for the pseudo-ROC analysis displayed in Figure 9 are ranked and cut-offs for the three bins are chosen based on the jumps visible at 0.86 and 0.89. [file 1471-2164-11-349-S7.PDF]
