# Supplementary material for: Comparison of normalization methods for Illumina BeadChip HumanHT-12 v3
Source: BMC Genomics. 2010 Jun 2;11:349. doi: 10.1186/1471-2164-11-349 (PMC3091625; doi:10.1186/1471-2164-11-349)

bg_average (-1)

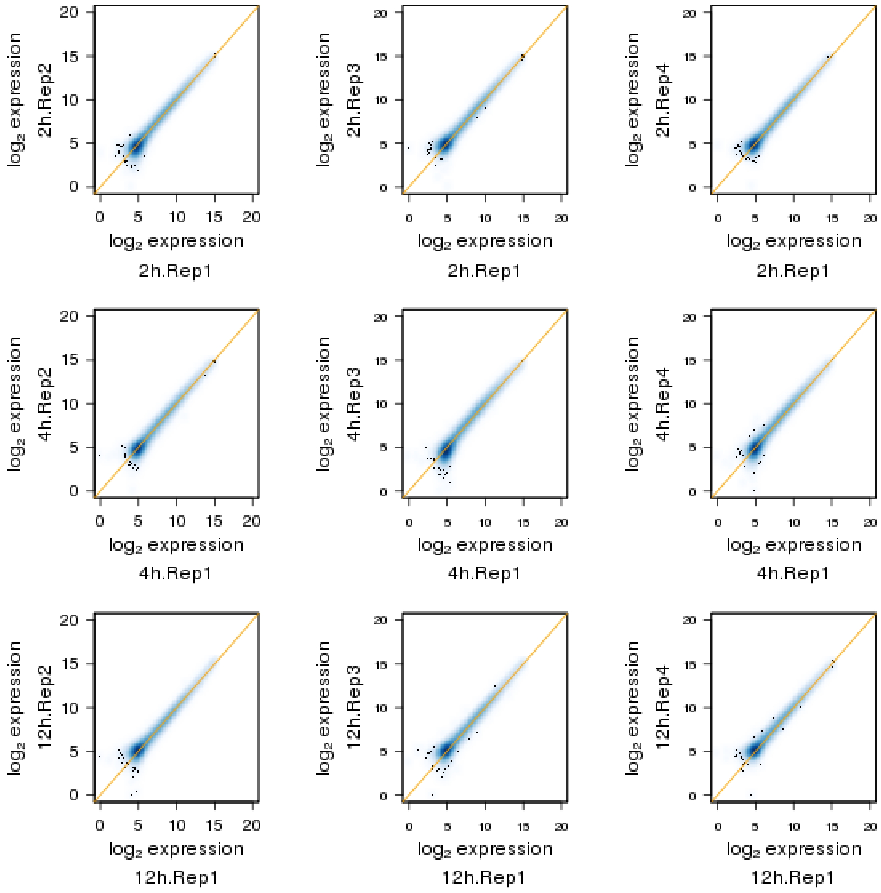

bg_cubicSpline (-1)

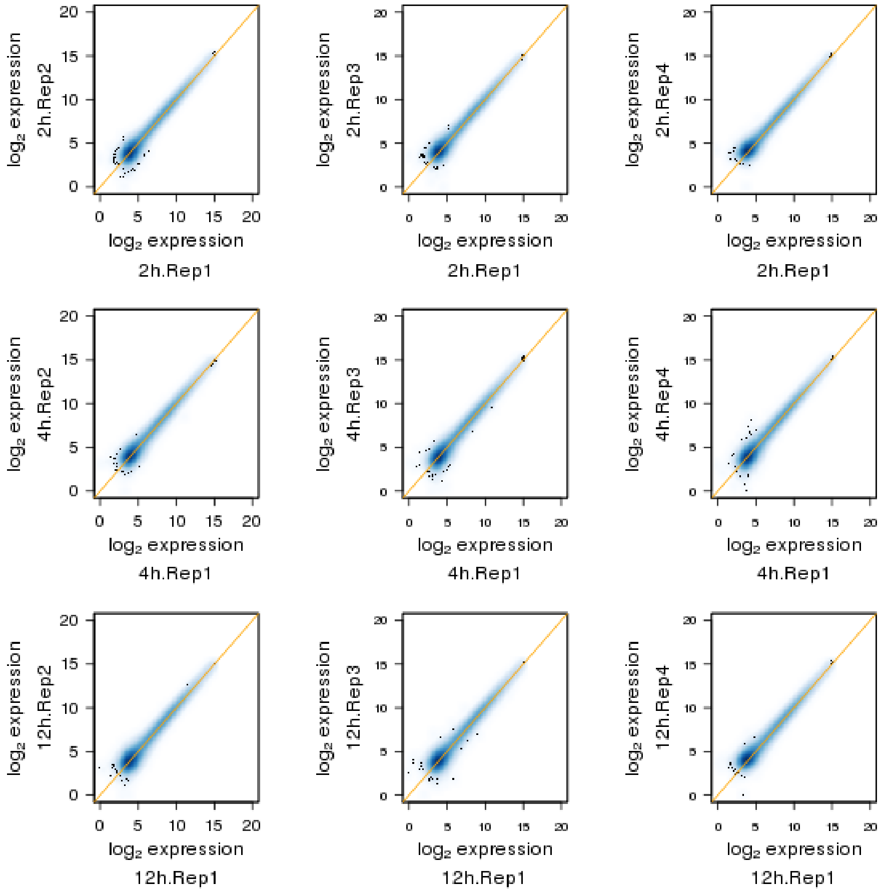

bg_forcePos_log_loess (0)

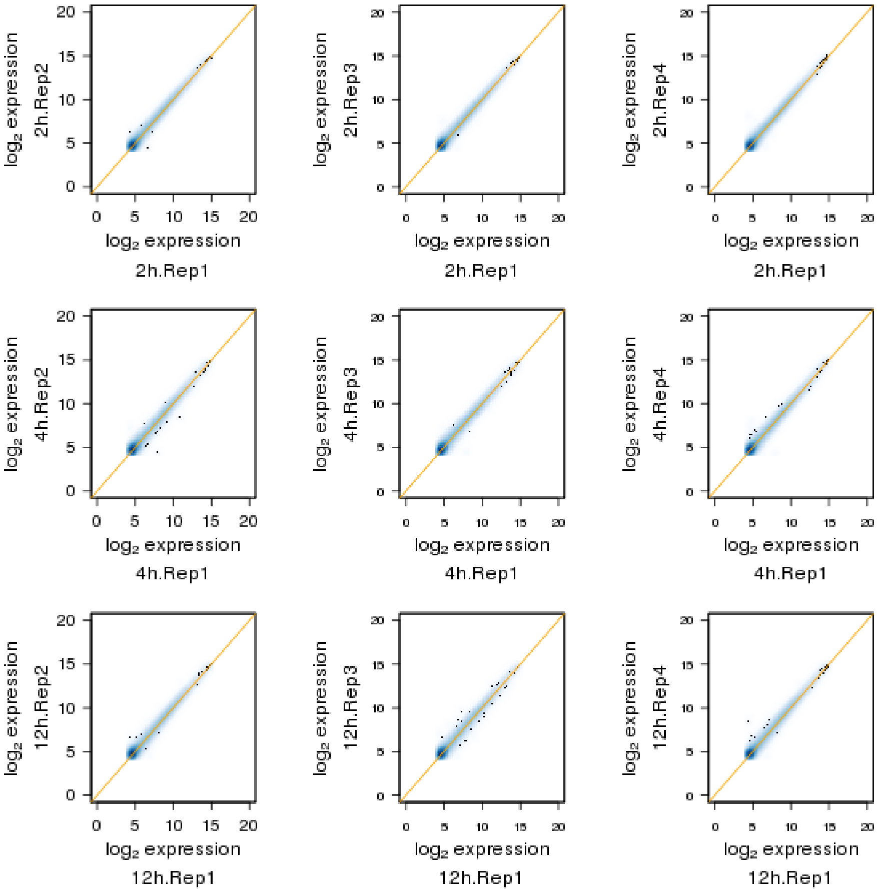

bg_forcePos_log_quantile (-1)

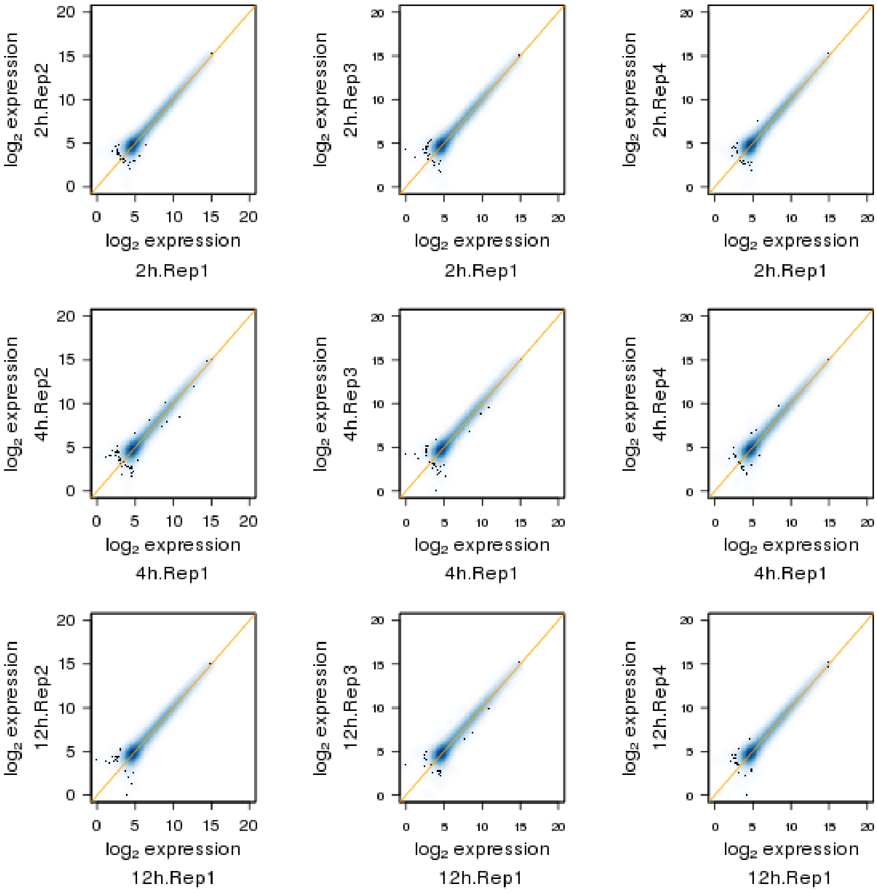

bg_forcePos_log_rsn (-1)

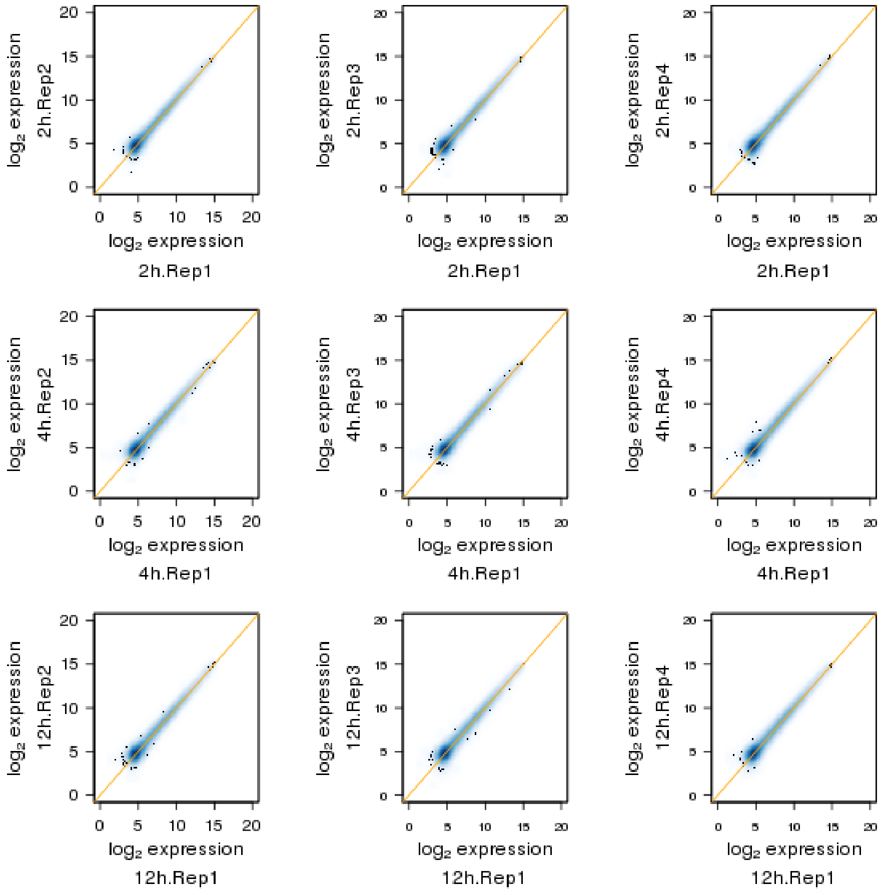

bg_noNorm (-1)

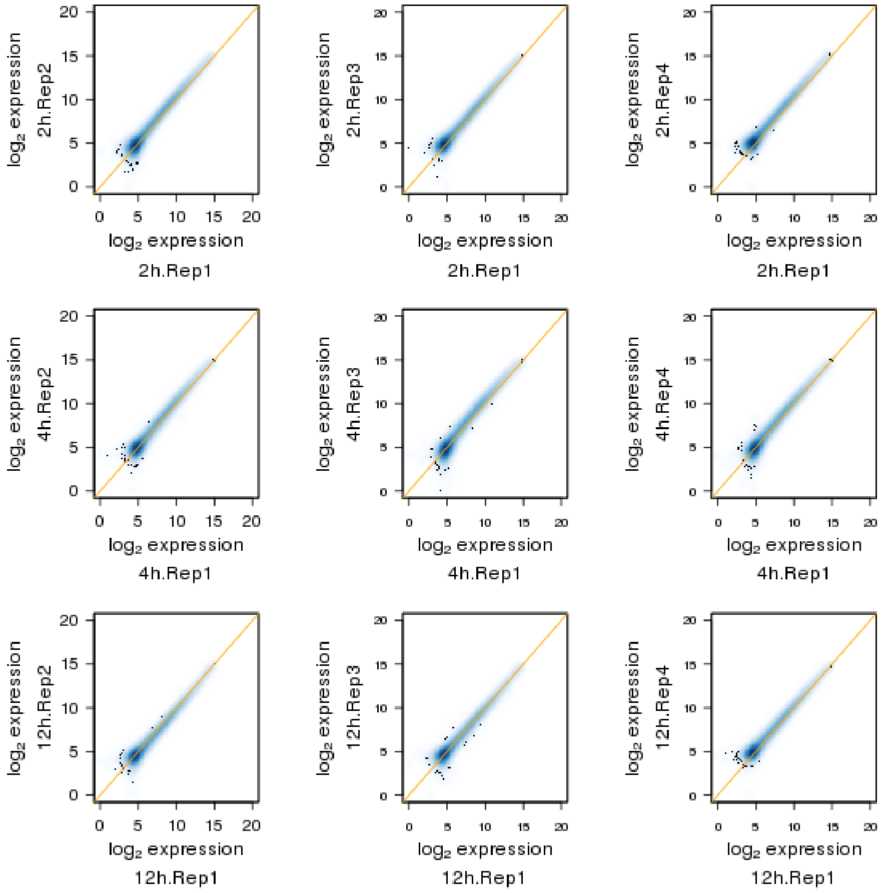

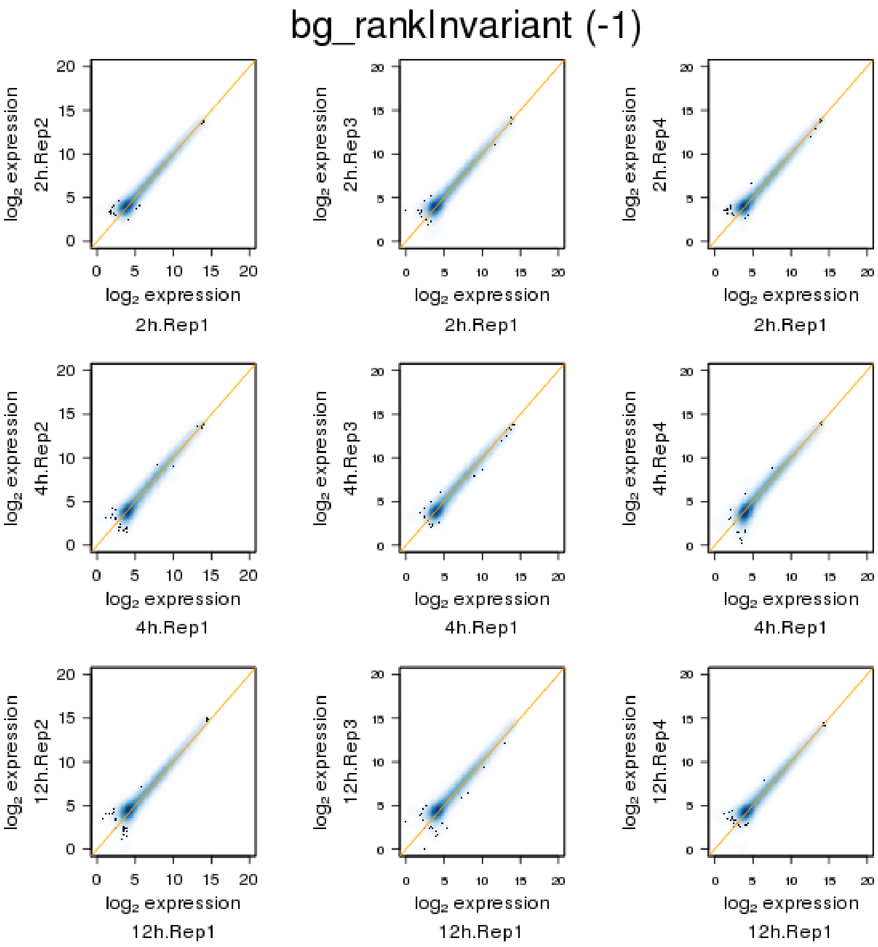
bg_rankInvariant (-1)
log2 expression 2h.Rep2
log2 expression 2h.Rep3
log2 expression 2h.Rep4
log2 expression 2h.Rep1
log2 expression 4h.Rep2
log2 expression 4h.Rep3
log2 expression 4h.Rep4
log2 expression 4h.Rep1
log2 expression 12h.Rep2
log2 expression 12h.Rep3
log2 expression 12h.Rep4
log2 expression 12h.Rep1
0
5
10
15
20

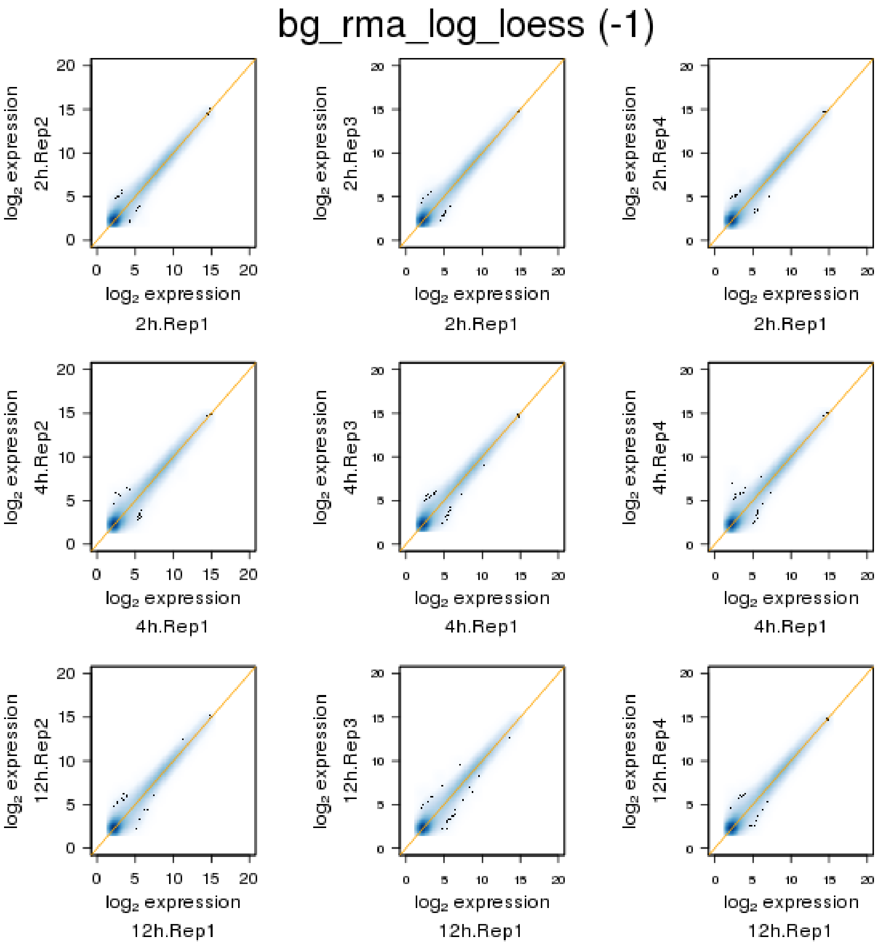
bg_rma_log_loess (-1)
log2 expression 2h.Rep2
log2 expression 2h.Rep3
log2 expression 2h.Rep4
log2 expression 2h.Rep1
log2 expression 4h.Rep2
log2 expression 4h.Rep3
log2 expression 4h.Rep4
log2 expression 4h.Rep1
log2 expression 12h.Rep2
log2 expression 12h.Rep3
log2 expression 12h.Rep4
log2 expression 12h.Rep1
0
5
10
15
20

bg_rma_log_quantile (-2)

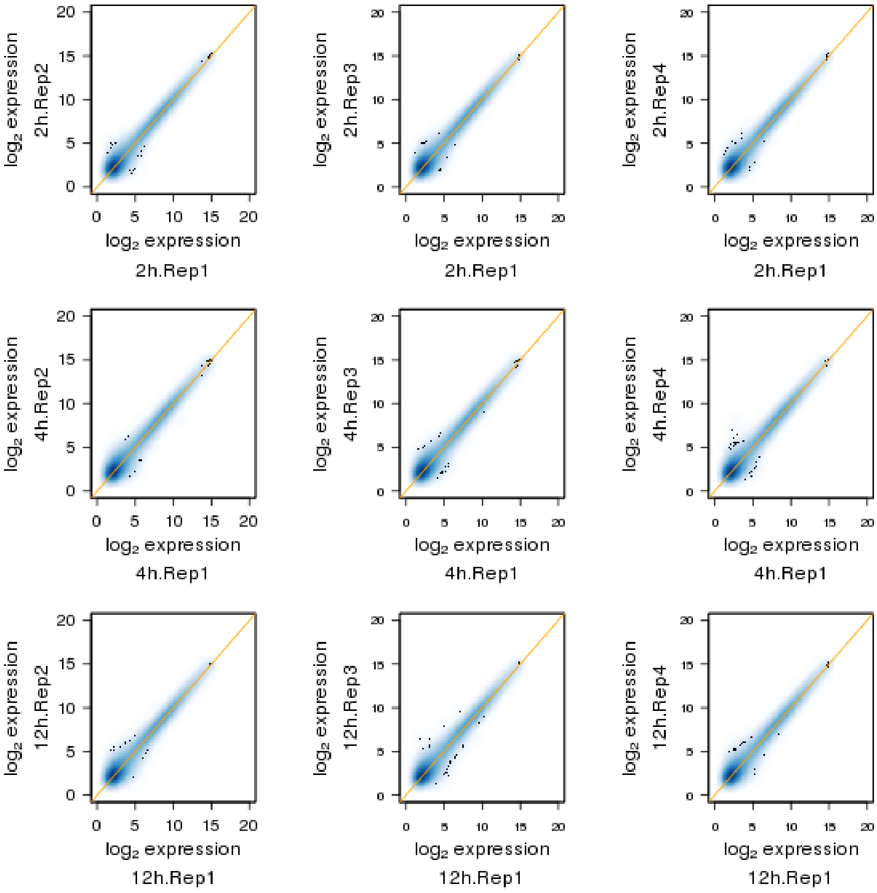

bg_rma_log_rsn (-2)

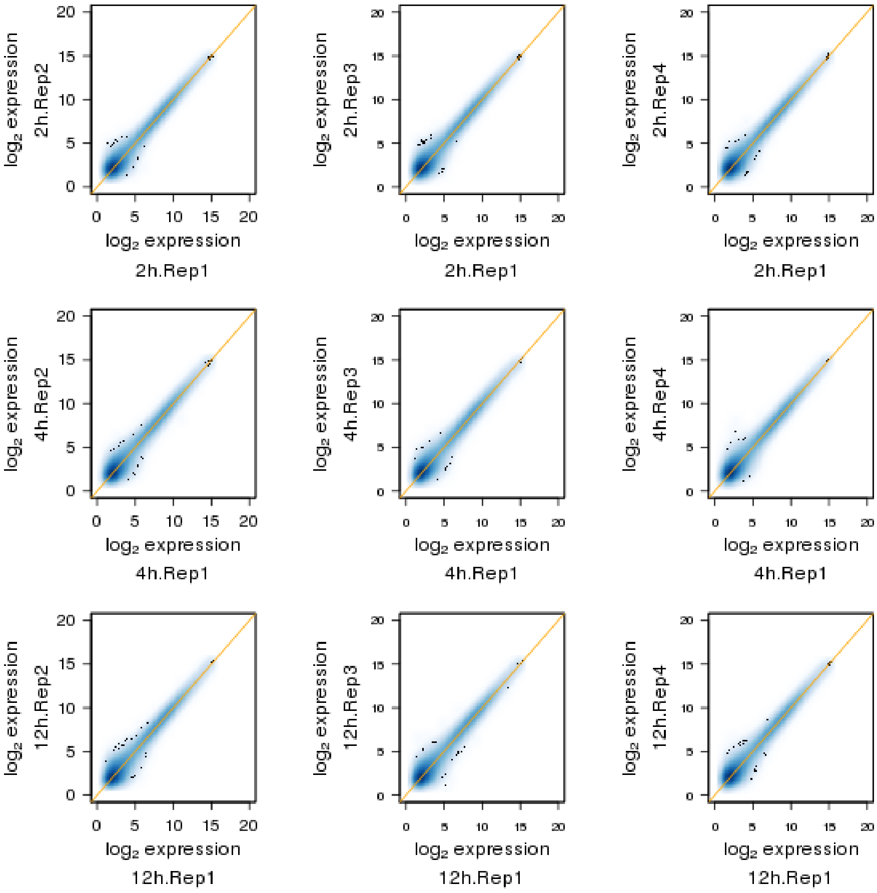

bg_vsn (-2)

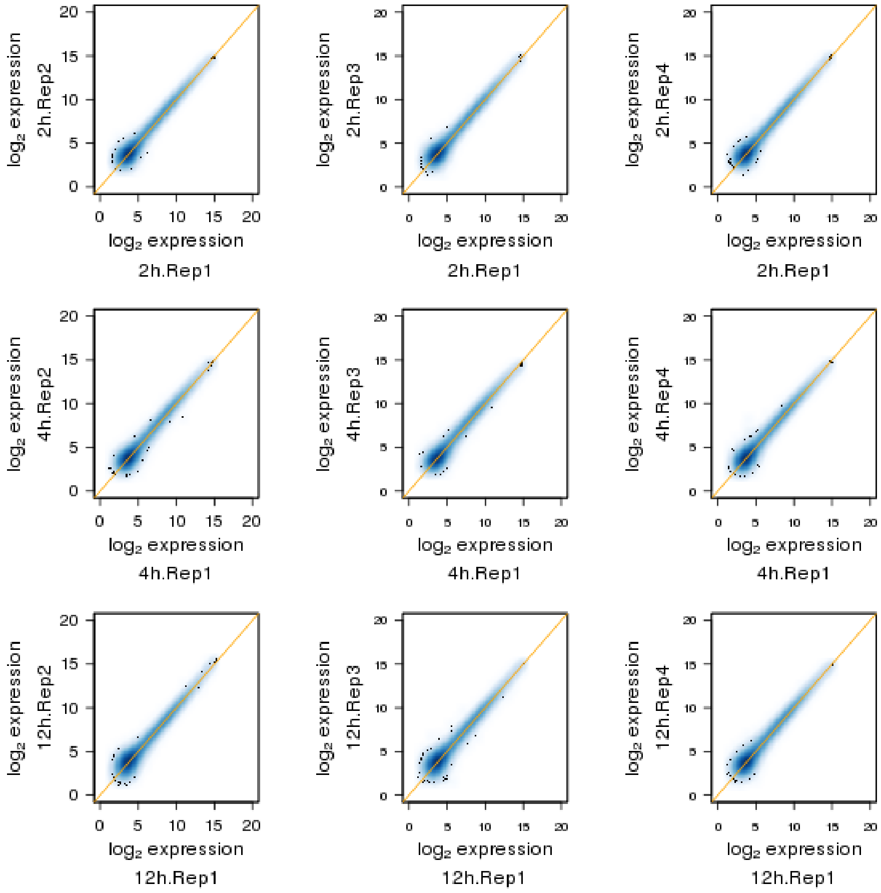

bg_vst_loess (1)

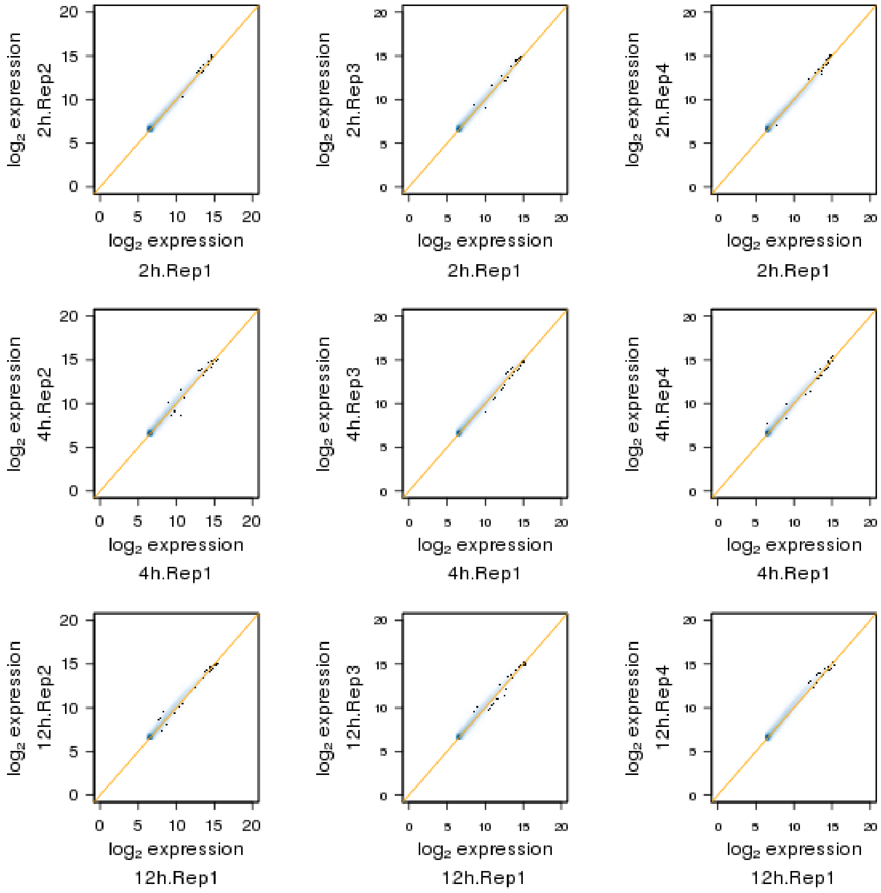

bg_vst_quantile (2)

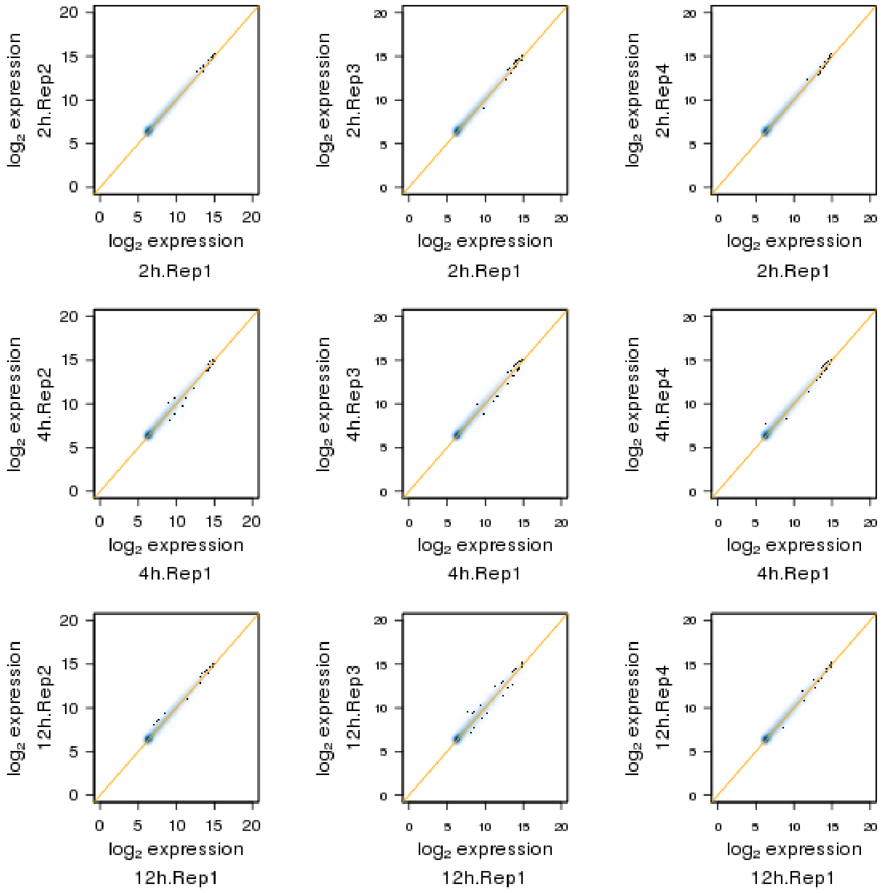

bg_vst_rsn (2)

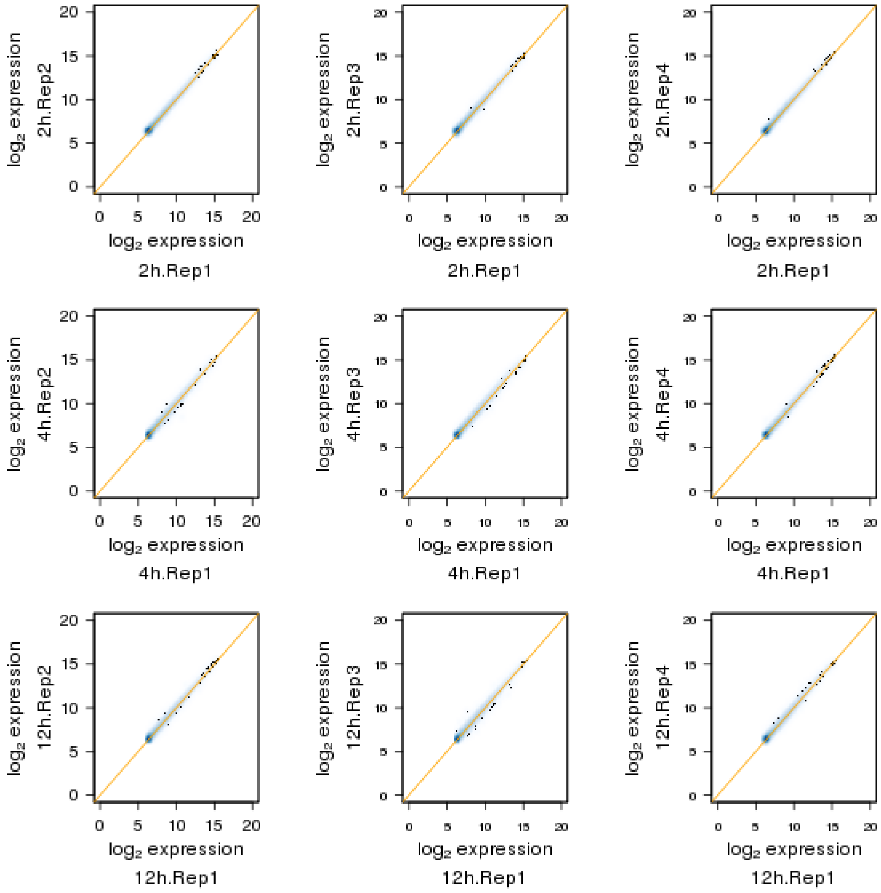

noBg_average (1)

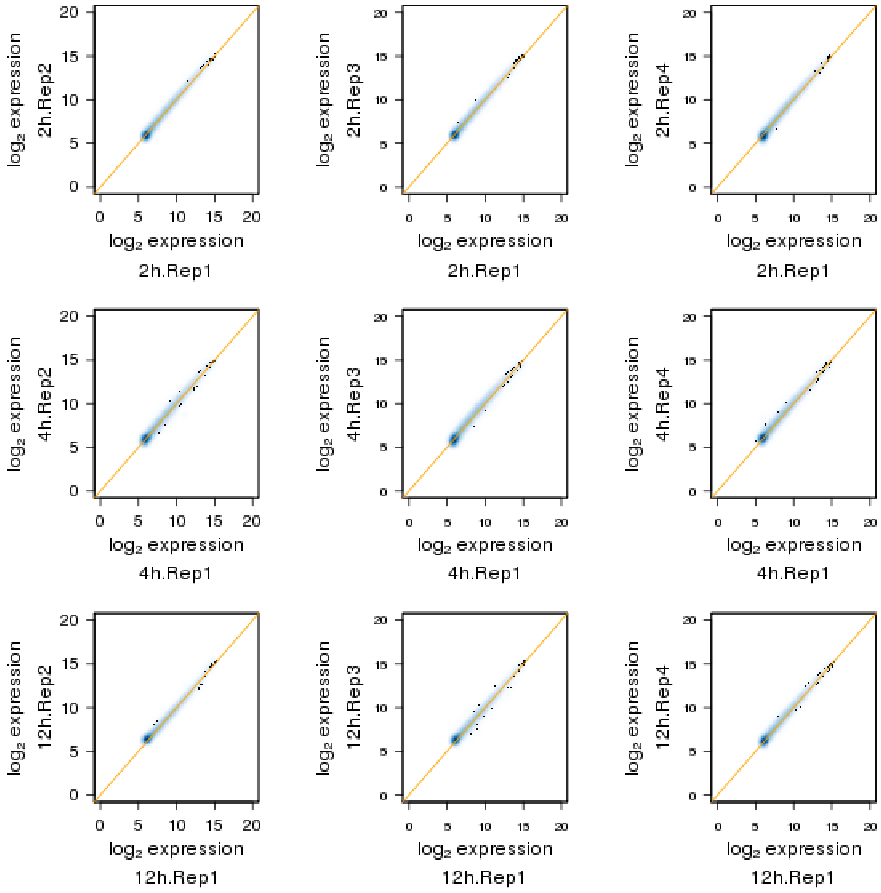

noBg_cubicSpline (2)

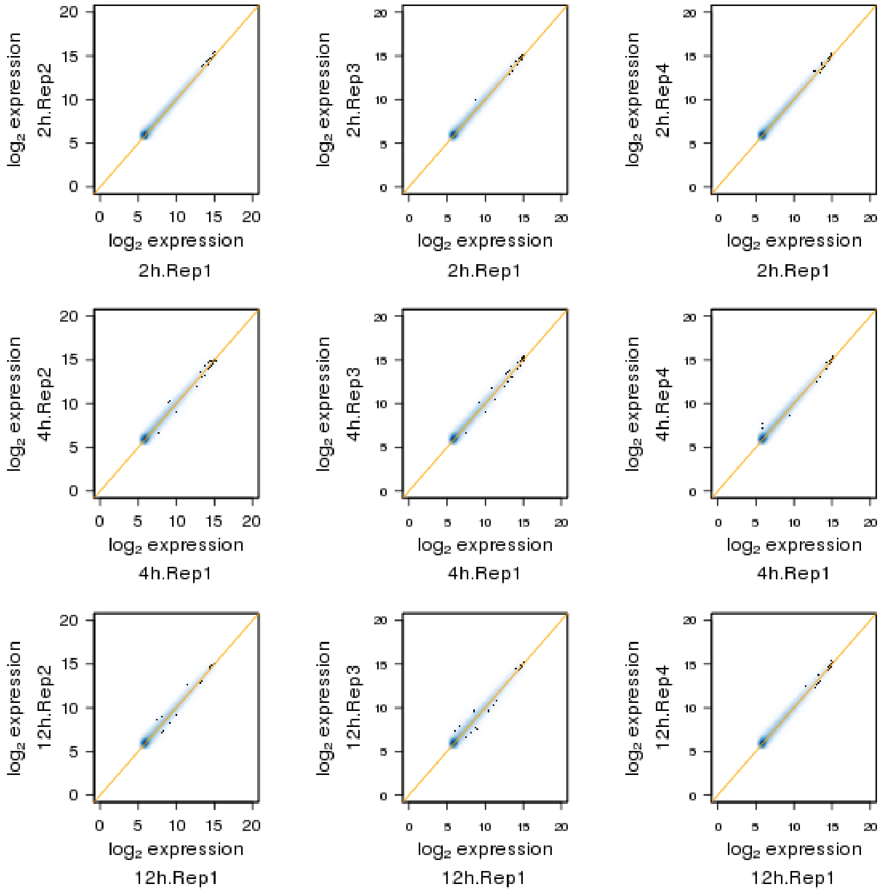

noBg_log_loess (1)

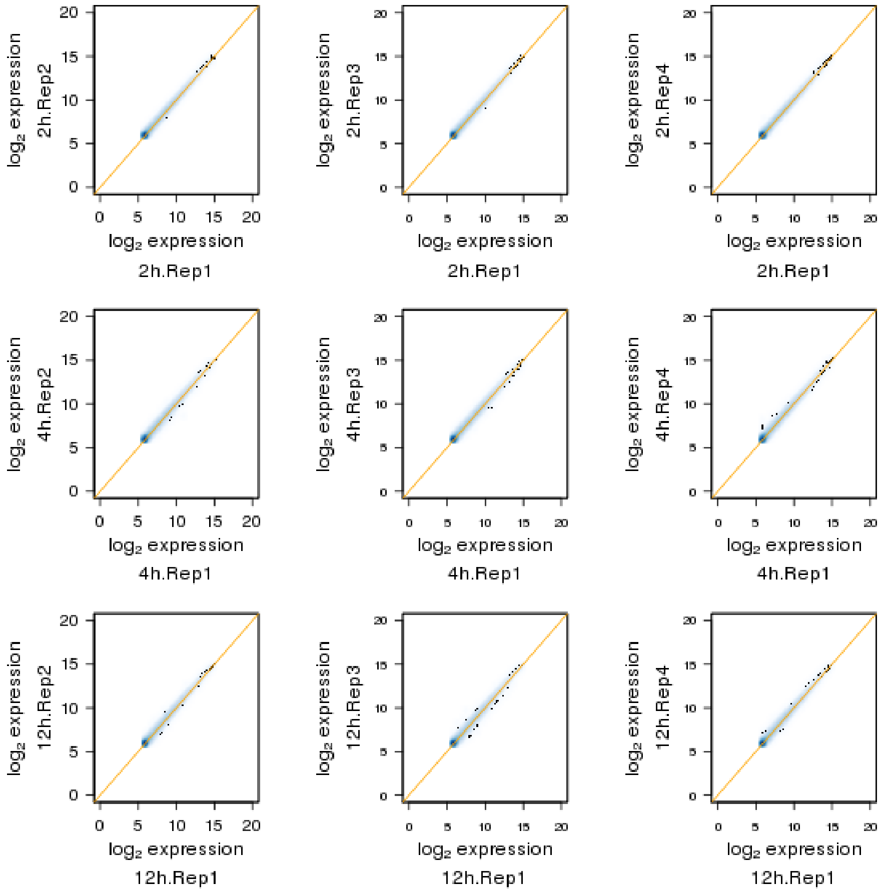

noBg_log_quantile (1)

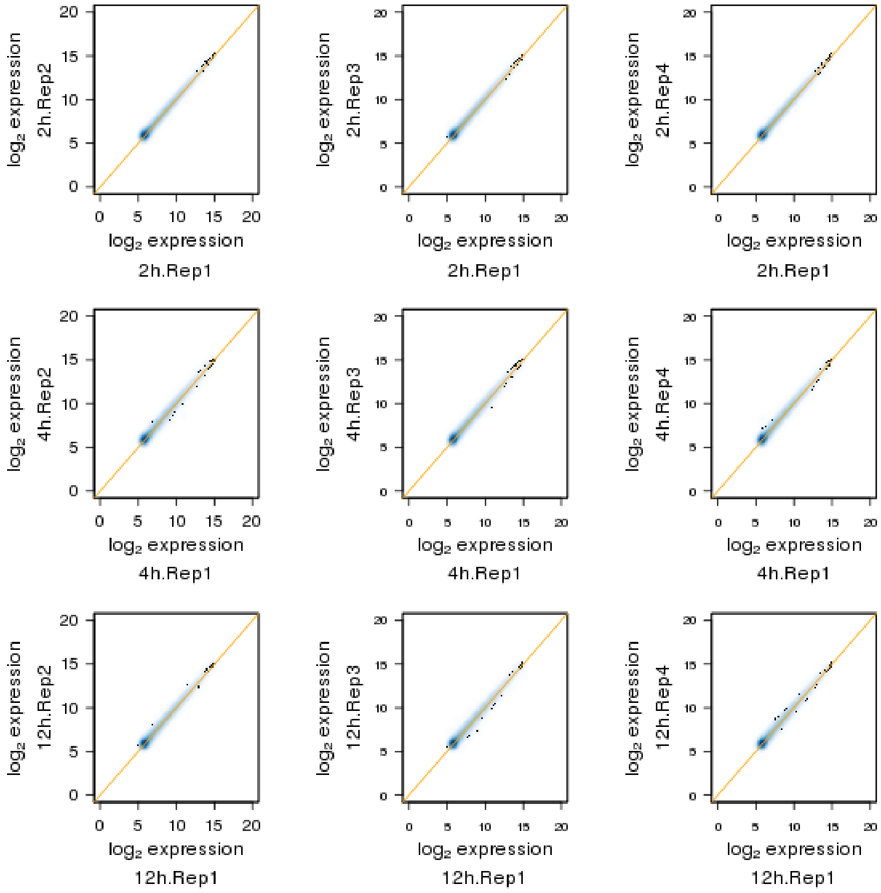

noBg_log_rsn (1)

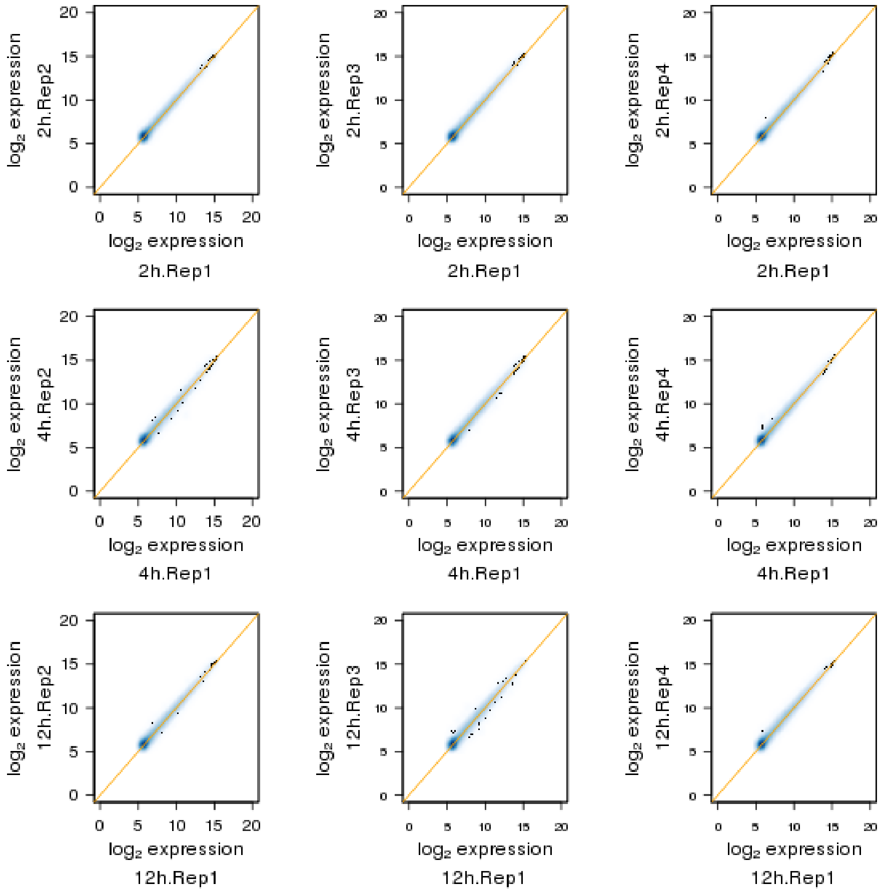

noBg_noNorm (1)

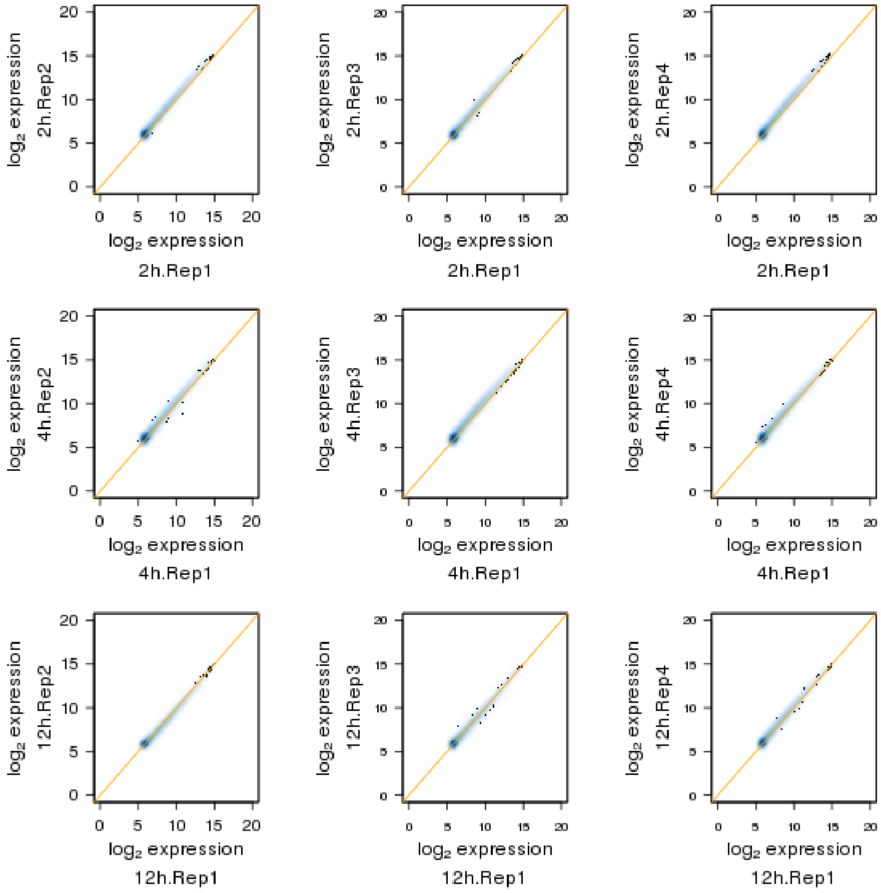

noBg_rankInvariant (2)

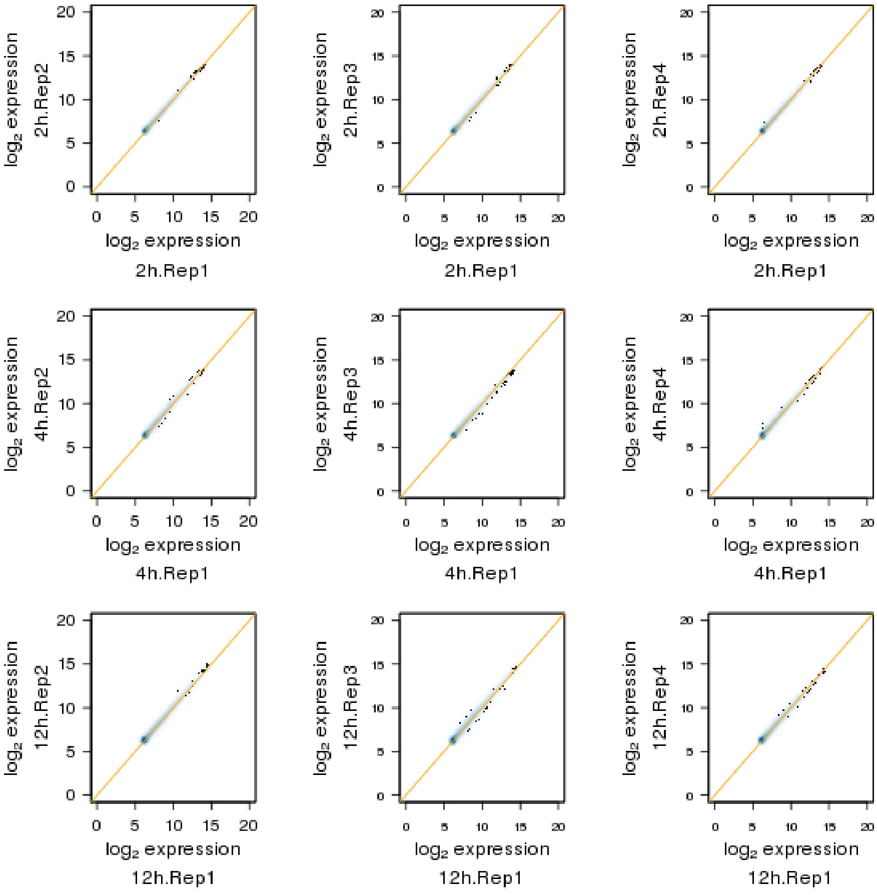

noBg_vsn (1)

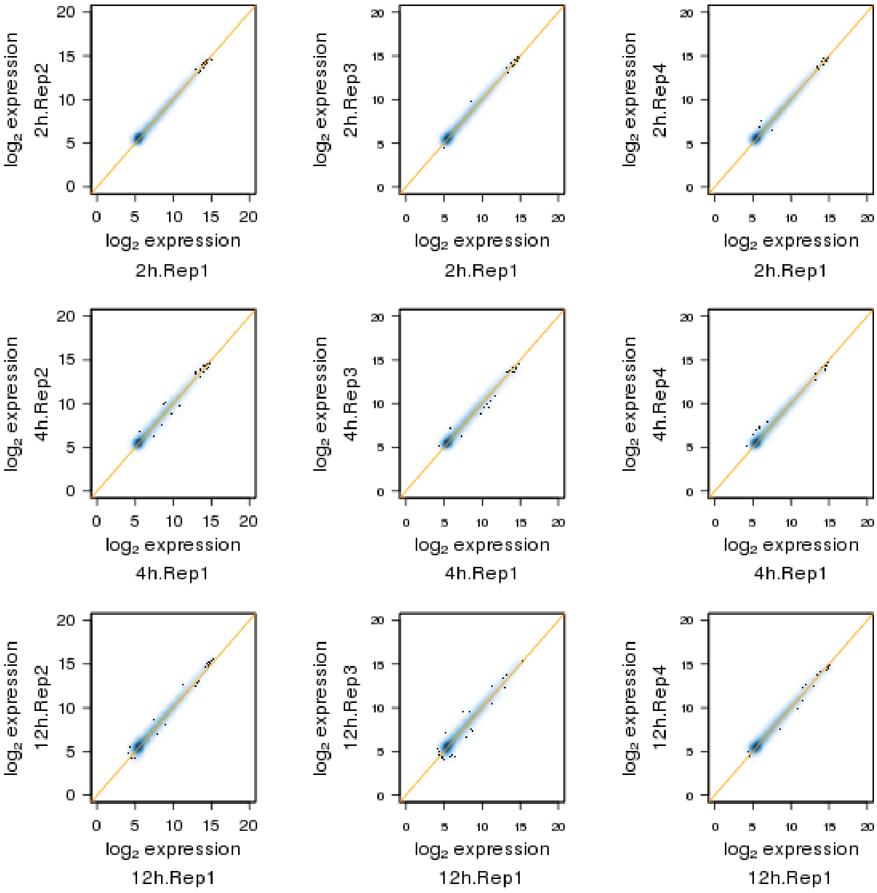

noBg_vst_loess (2)

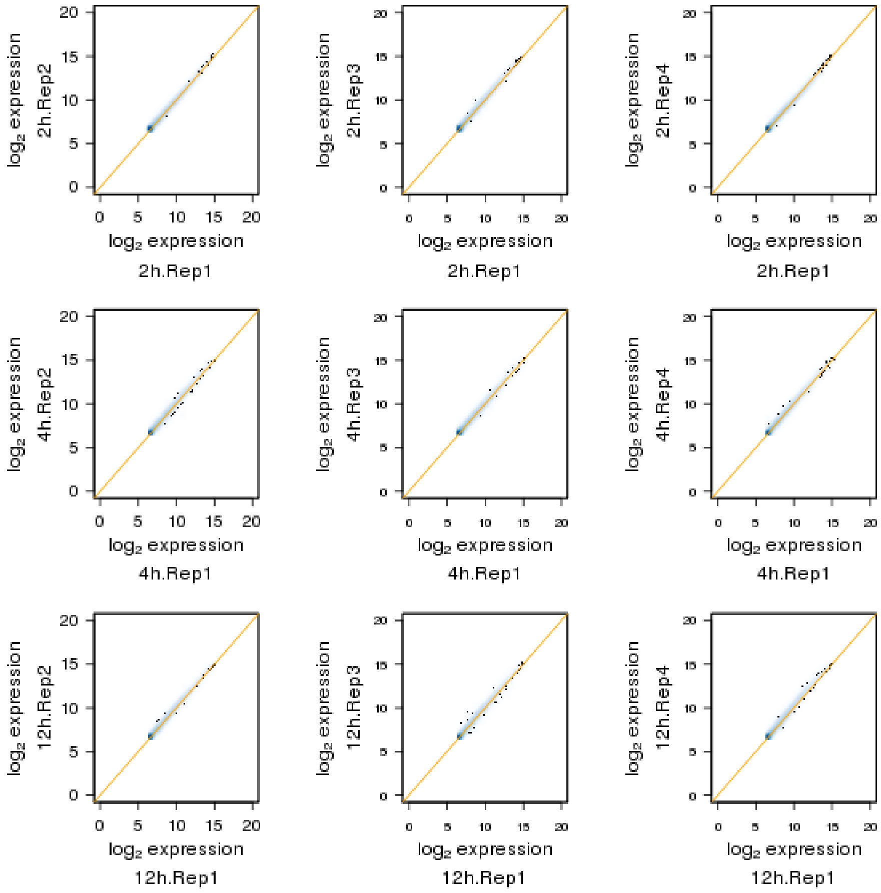

noBg_vst_quantile (2)

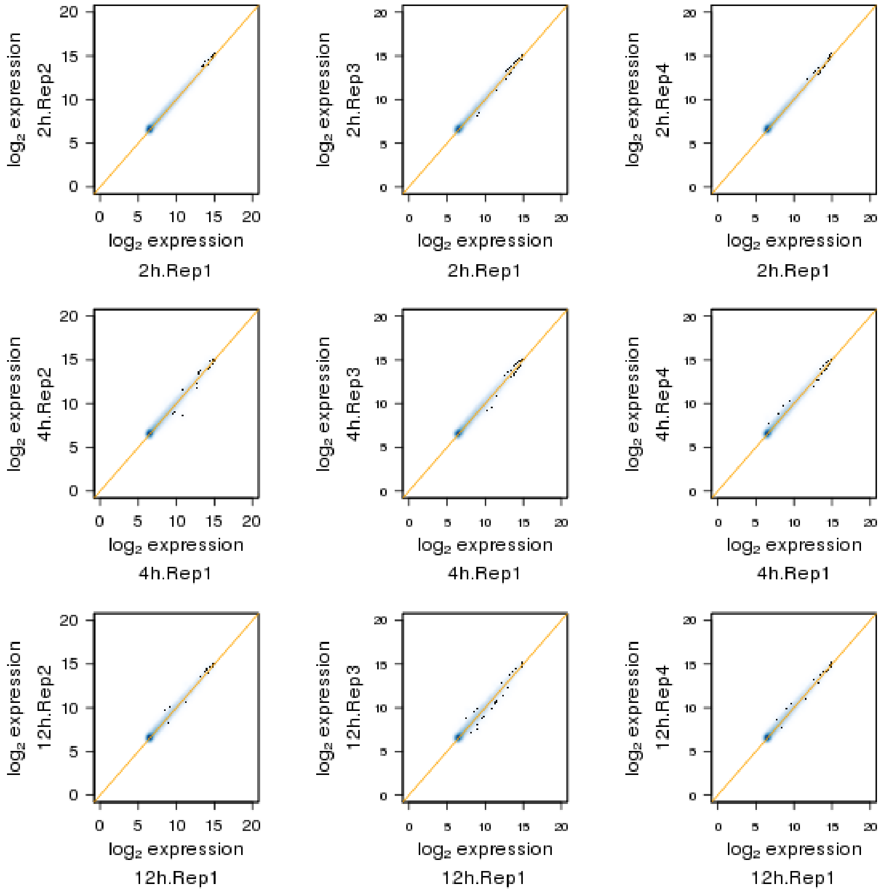

noBg_vst_rsn (2)

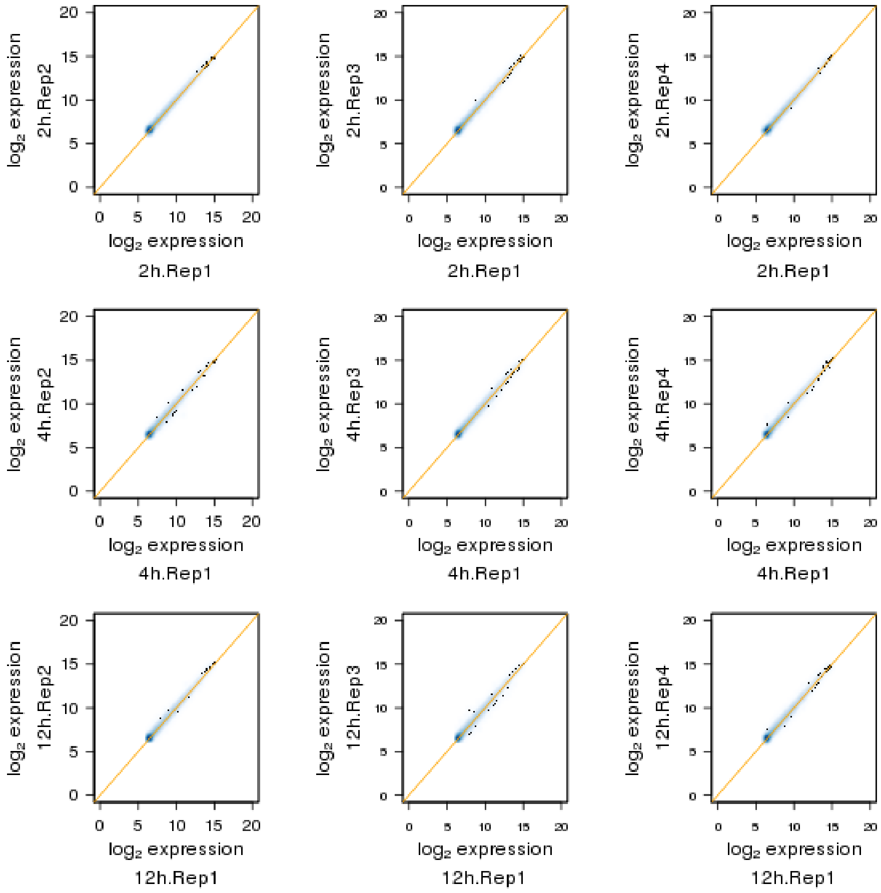

Supplement: Additional file 6 — Scatterplots between replicates. After application of different normalization methods, expression values for the replicates are plotted against each other. The orange line indicates the main diagonal. [file 1471-2164-11-349-S6.PDF]
