# Supplementary material for: Comparison of normalization methods for Illumina BeadChip HumanHT-12 v3
Source: BMC Genomics. 2010 Jun 2;11:349. doi: 10.1186/1471-2164-11-349 (PMC3091625; doi:10.1186/1471-2164-11-349)
Supplement: Additional file 9 — Orthogonal regression between qRT-PCR and normalization based log2 ratios. Regression of log2 ratios based on different normalization methods (y-axis) against qRT-PCR log2 ratios (x-axis). Equations and the respective regression lines are displayed in red. The grey dashed line indicates the main diagonal. [file 1471-2164-11-349-S9.PDF]

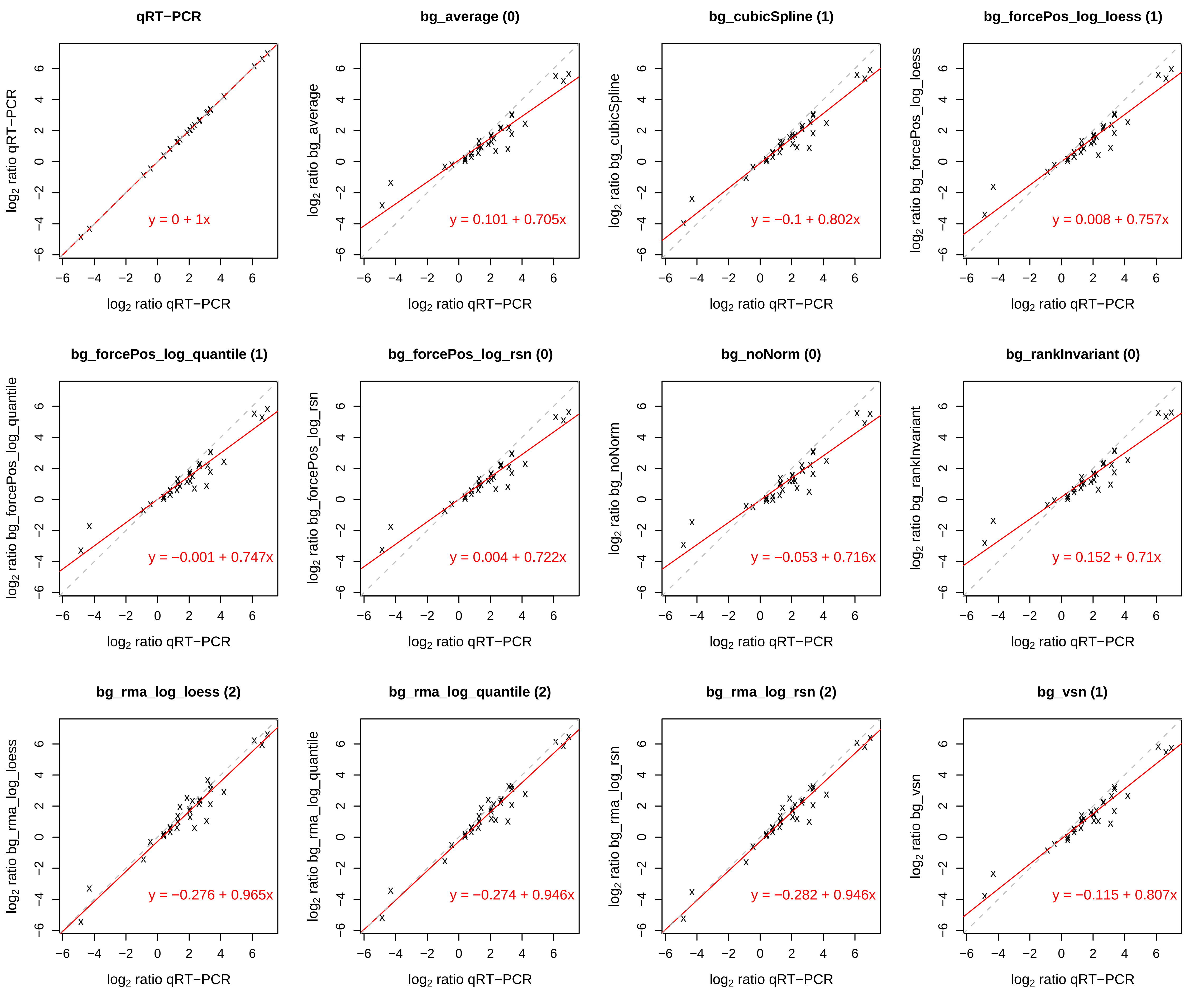
qRT−PCR
log2 ratio qRT−PCR
y = 0 + 1x
bg_average (0)
log2 ratio bg_average
y = 0.101 + 0.705x
bg_cubicSpline (1)
log2 ratio bg_cubicSpline
y = −0.1 + 0.802x
bg_forcePos_log_loess (1)
log2 ratio bg_forcePos_log_loess
y = 0.008 + 0.757x
bg_forcePos_log_quantile (1)
log2 ratio bg_forcePos_log_quantile
y = −0.001 + 0.747x
bg_forcePos_log_rsn (0)
log2 ratio bg_forcePos_log_rsn
y = 0.004 + 0.722x
bg_noNorm (0)
log2 ratio bg_noNorm
y = −0.053 + 0.716x
bg_rankInvariant (0)
log2 ratio bg_rankInvariant
y = 0.152 + 0.71x
bg_rma_log_loess (2)
log2 ratio bg_rma_log_loess
y = −0.276 + 0.965x
bg_rma_log_quantile (2)
log2 ratio bg_rma_log_quantile
y = −0.274 + 0.946x
bg_rma_log_rsn (2)
log2 ratio bg_rma_log_rsn
y = −0.282 + 0.946x
bg_vsn (1)
log2 ratio bg_vsn
y = −0.115 + 0.807x
log2 ratio qRT−PCR

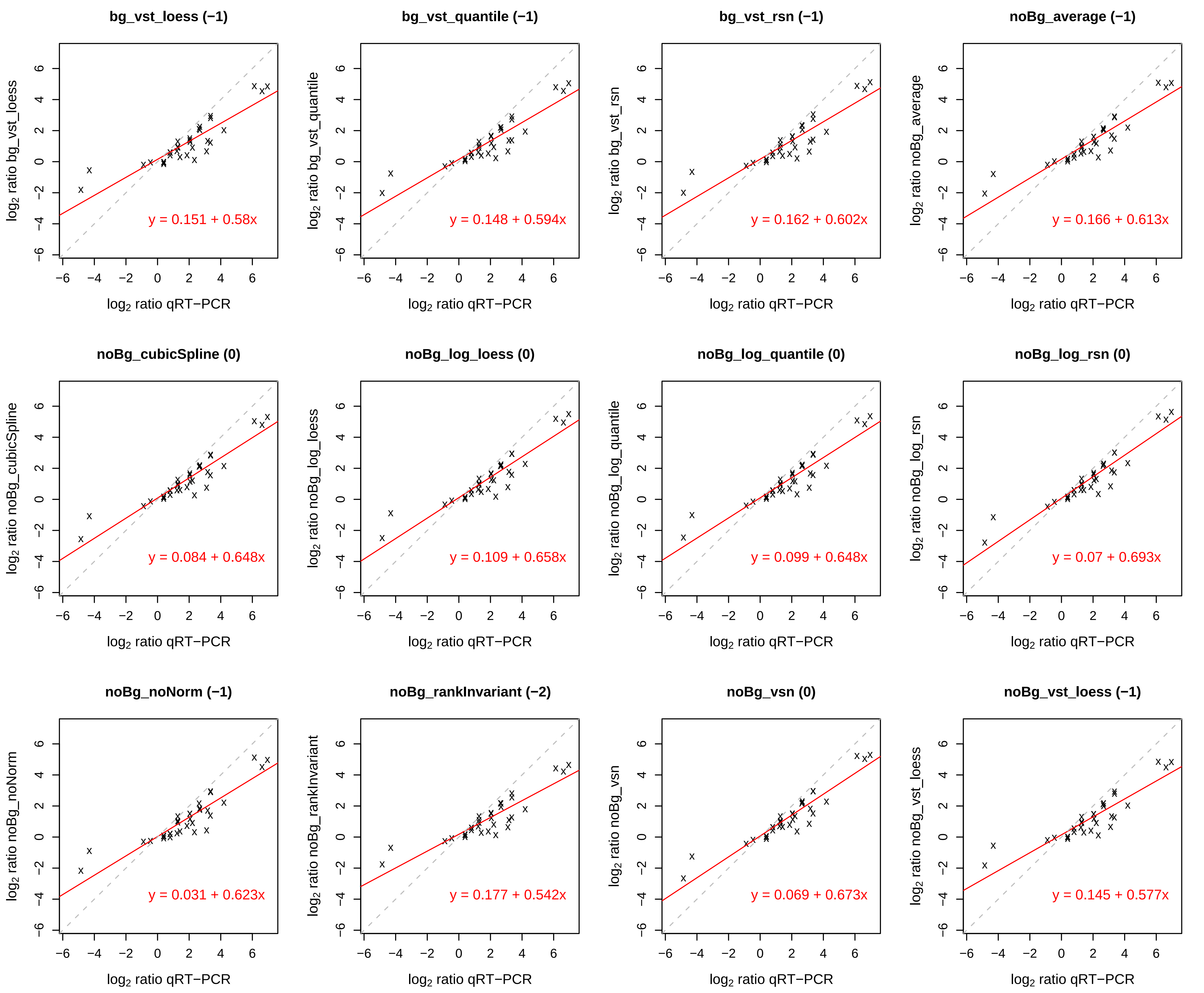
bg_vst_loess (−1)
y = 0.151 + 0.58x
log2 ratio bg_vst_loess
bg_vst_quantile (−1)
y = 0.148 + 0.594x
log2 ratio bg_vst_quantile
bg_vst_rsn (−1)
y = 0.162 + 0.602x
log2 ratio bg_vst_rsn
noBg_average (−1)
y = 0.166 + 0.613x
log2 ratio noBg_average
noBg_cubicSpline (0)
y = 0.084 + 0.648x
log2 ratio noBg_cubicSpline
noBg_log_loess (0)
y = 0.109 + 0.658x
log2 ratio noBg_log_loess
noBg_log_quantile (0)
y = 0.099 + 0.648x
log2 ratio noBg_log_quantile
noBg_log_rsn (0)
y = 0.07 + 0.693x
log2 ratio noBg_log_rsn
noBg_noNorm (−1)
y = 0.031 + 0.623x
log2 ratio noBg_noNorm
noBg_rankInvariant (−2)
y = 0.177 + 0.542x
log2 ratio noBg_rankInvariant
noBg_vsn (0)
y = 0.069 + 0.673x
log2 ratio noBg_vsn
noBg_vst_loess (−1)
y = 0.145 + 0.577x
log2 ratio noBg_vst_loess
log2 ratio qRT−PCR

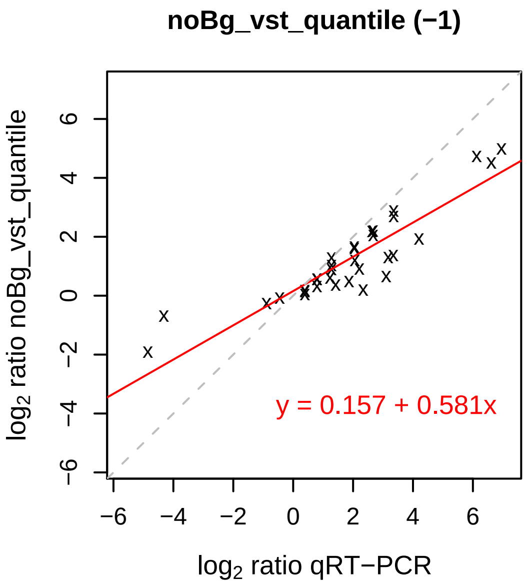

noBg_vst_quantile (−1)
log2 ratio noBg_vst_quantile
log2 ratio qRT−PCR
y = 0.157 + 0.581x

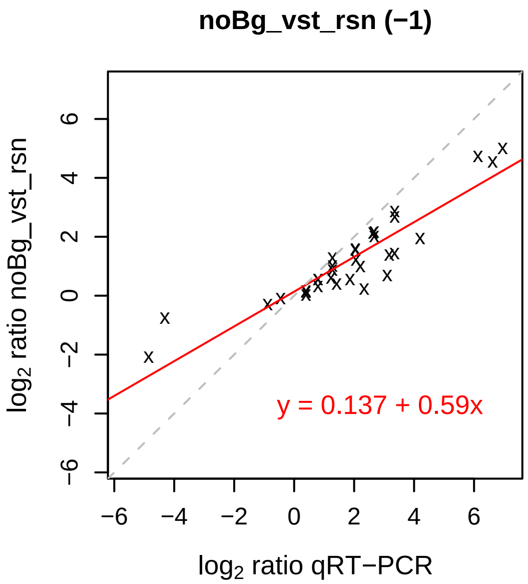

noBg_vst_rsn (−1)
log2 ratio noBg_vst_rsn
log2 ratio qRT−PCR
y = 0.137 + 0.59x
